# High-efficiency methane consumption by atmospheric methanotrophs in subsurface karst caves: The irrefutable methane sink

**DOI:** 10.1126/sciadv.ady5942

**Published:** 2026-02-04

**Authors:** Xiaoyan Liu, Xiaoyu Cheng, Yiming Zhang, Rui Zhao, Weiqi Wang, Yang Li, Zhong-Qiang Chen, Xincheng Qiu, Olli H. Tuovinen, Ian D. Bull, Richard P. Evershed, Hongmei Wang

**Affiliations:** ^1^ State Key Laboratory of Geomicrobiology and Environmental Change, School of Environmental Studies, China University of Geosciences, Wuhan 430078, China.; ^2^ School of Chemistry, University of Bristol, Cantock’s Close, Bristol BS8 1TS, UK.; ^3^ Department of Earth, Atmospheric, and Planetary Sciences, Massachusetts Institute of Technology, Cambridge, MA 02139, USA.; ^4^ Department of Microbiology, The Ohio State University, Columbus, OH 43210, USA.

## Abstract

Subsurface karst systems represent substantial but underexplored methane sinks, yet the identities and activities of cave-dwelling methanotrophs remain poorly characterized. We detected increased methane oxidation rates from 2.9 ± 0.1 to 90.7 ± 4.5 ng·g^−1^·hour^−1^ while supplied with 2 to 500 parts per million (ppm) CH_4_ to cave sediments. Atmospheric methanotroph Upland Soil Clusters γ (USCγ), responsible for this oxidation, was further assigned to three genera within the family *Candidatus* (*Ca*.) Methyloligotrophaceae, including two previously unrecognized genera. Nano-scale secondary ion mass spectrometry (NanoSIMS) imaging and the produced ^13^C-PLFAs (phospholipid fatty acids) and ^13^CO_2_ in ^13^CH_4_-fed microcosm confirmed methane as both carbon and energy sources. These methanotrophs exhibited low half-saturation constant (*K*_m_; 138.8 ± 15.8 ppm), high carbon assimilation efficiency (>50%), and metabolic versatility, as revealed by metagenomics and metatranscriptomics analyses. By extrapolating global distribution of *Ca.* Methyloligotrophaceae and comparing methane oxidation rates between caves and soil ecosystems, we conservatively estimate that subsurface karst in southwest China sequester ~0.56 Tg CH_4_ annually. These findings highlight the ecological importance of karst ecosystems as a previously overlooked methane sink.

## Teaser

Microbes in subsurface caves consume trace amount of methane from the air.

## INTRODUCTION

Methane (CH_4_) is a potent greenhouse gas with a global warming potential over 25 times greater than carbon dioxide (CO_2_) over a 100-year period, making it a critical component in global climate regulation (*1*). Despite extensive natural and anthropogenic sources of methane, its atmospheric concentration is substantially moderated by microbial methane oxidation, predominantly mediated by methane-oxidizing bacteria (MOB) or methanotroph, which act as a primary biological sink (*2*). Soil, especially through the activity of MOB, is estimated to consume ~30 Tg of methane from the atmosphere annually, underscoring their pivotal role in the global methane cycle (*3*). However, mounting evidence suggests that climate change and anthropogenic pressures are destabilizing soil microbial communities, raising concerns about the long-term resilience of soils as effective methane sinks (*4*, *5*).

Amid these uncertainties, there has been increasing effort to identify, quantify, and understand existing and relatively stable methane sinks more comprehensively. Subsurface karst cave, characterized by lower CH_4_ levels than ambient air, climatic stability, limited anthropogenic disturbance, and low-energy oligotrophic conditions, have emerged as promising yet underexplored natural ecosystems where microbial methane oxidation may occur, potentially contributing to a more accurate assessment of the global methane budget and its resilience to disturbance (*6*–*8*). Studies consistently report near-universal methane depletion in cave atmospheres relative to the outside air, suggesting that active methane oxidation processes are at play (*6*, *7*). Although early hypotheses emphasized abiotic oxidation via hydroxyl radicals or radiolytic reactions driven by the decay of radon-222 (*7*), an expanding body of evidence points toward microbial mediation. This is supported by observed CH_4_ and CO_2_ dynamics across multiple caves, distinct isotopic signatures (δ^13^C_CH4_, δ^2^H_CH4_, and δ^13^C_CO2_), and laboratory incubations demonstrating greater methane consumption in live cave sediments compared to sterilized controls (*6*, *8*–*13*).

Despite this growing recognition, the identity, metabolic strategies, and ecological functions of the cave-associated methanotrophs remain largely unresolved. Among the diverse MOB lineages, atmospheric methane-oxidizing bacteria (atmMOB) are particularly relevant in cave settings, where methane levels often fall below atmospheric concentration (*11*, *14*). Within this group, the Upland Soil Clusters (USC), comprising USCα and USCγ, represents key clades adapted to trace-methane conditions. While USCα, affiliated with type II MOB (Alphaproteobacteria), is well characterized due to the isolation of *Methylocapsa gorgona* MG08 (*15*, *16*), its counterpart USCγ, remains poorly understood. USCγ belongs to the Gammaproteobacteria and is phylogenetically closed to type Ib MOB, although some alternative classifications propose its affiliation with type Ic or type Id MOB (*17*, *18*). USCγ is frequently detected in neutral to alkaline, nutrient-poor soils, such as grasslands, where it often dominates methanotrophic communities under low-methane conditions (*19*–*21*). The ecological prominence of USCγ in terrestrial systems highlights its broader significance in methane-limited environments.

Among the methane-limited environments, alkaline karst caves stand out as particularly notable habitats because they serve as an accessible and climatically stable subterranean ecosystem, providing a rare opportunity to study methanotrophy under extreme oligotrophic and methane-poor conditions. In such caves, USCγ is increasingly recognized as the dominant methanotroph and comprises a substantial fraction of microbial communities, ranging from 2 to 12% in several Australian caves to up to 20% in Heshang Cave (Central China) and up to 6.25% in over 20 North America caves (*11*, *14*, *22*, *23*). However, only one metagenome-assembled genome (MAG) of USCγ has been reported to date, revealing limited metabolic information. This MAG shares >90% 16*S* ribosomal RNA (rRNA) identity with Chromatiales bacteria and harbors genes for methane oxidation to CO_2_, but notably lacks formaldehyde dehydrogenase while having the serine biosynthesis pathway that underpins the serine cycle for formaldehyde assimilation (*24*). This discrepancy between the ecological prominence of USCγ and the paucity of genomic and physiological information highlights a critical knowledge gap. Thus, a deeper understanding of USCγ’s metabolic strategies is essential not only to clarify its ecological role in caves but also to evaluate its broader significance in mitigating atmospheric methane.

In this study, by combining field observations with laboratory-based sediment incubations from Chang Cave, a pristine karst cave system, we examine the methane oxidation capacity and metabolic traits of USCγ, shedding light on its ecological functions across karst ecosystems in Southwest China. Through a multifaceted approach combining ^13^CH_4_-tracing microcosms, nanoscale secondary ion mass spectrometry (NanoSIMS) mapping, δ^13^C measurements of phospholipid fatty acid (PLFA) and total organic carbon (TOC), and metagenomic and metatranscriptomic analyses, we aim to (i) quantify the methane oxidation capacity of USCγ under trace-methane conditions, referring specifically to low half-saturation constant (*K*_m_) atmMOB unless otherwise noted, (ii) delineate its phylogenetic identity, and (iii) uncover its metabolic pathways involved in methane consumption. Our findings deepen the understanding of the biogeochemical role of methanotrophs in subterranean ecosystems and highlight the potential contribution of karst caves, as widespread and stable subterranean environments, to the global methane sink. These insights call for a more explicit consideration of karst cave ecosystems in global methane cycling models.

## RESULTS

### Methane-derived carbon efficiently assimilated by microbiota

**Fig. 1.**
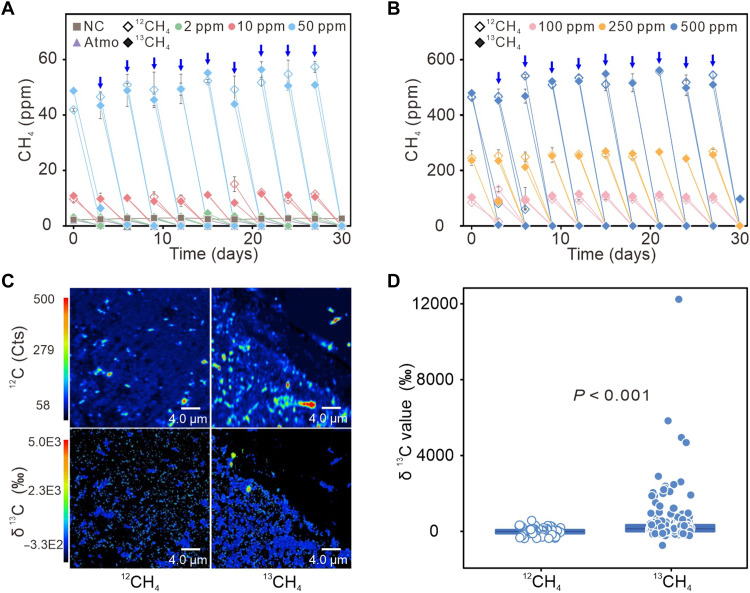
Methane concentration dynamics and carbon incorporation into single cells from cave sediments incubated with ^12^CH_4_ and ^13^CH_4_. (**A** and **B**) Variations in methane concentrations during incubation with ^12^CH_4_/^13^CH_4_ at concentrations ranging from 2 to 500 ppm [(A) 2, 10, and 50 ppm; (B) 100, 250, and 500 ppm]. NC, negative control; Atmo, atmospheric CH_4_ concentration. Blue arrows indicate the methane addition every 3 days to maintain target concentrations. (**C**) NanoSIMS imaging showing ^12^C counts (top) and carbon isotope ratio (δ^13^C; bottom) maps of single cells incubated with 500 ppm ^13^CH_4_ compared to 500 ppm ^12^CH_4_. Secondary ion counts are denoted as Cts in the figure. (**D**) Significant enrichment of ^13^C (*P* < 0.001) in individual cells incubated with 500 ppm ^13^CH_4_ after 30 days, in contrast to the ^12^CH_4_-exposed groups (with 88 and 387 cells detected in the ^12^CH_4_- and ^13^CH_4_-exposed groups, respectively). The difference in the number of cells shown reflects imaging field selection rather than true differences in cell abundance between treatments, and the results were confirmed with replicate analyses.

**Fig. 2.**
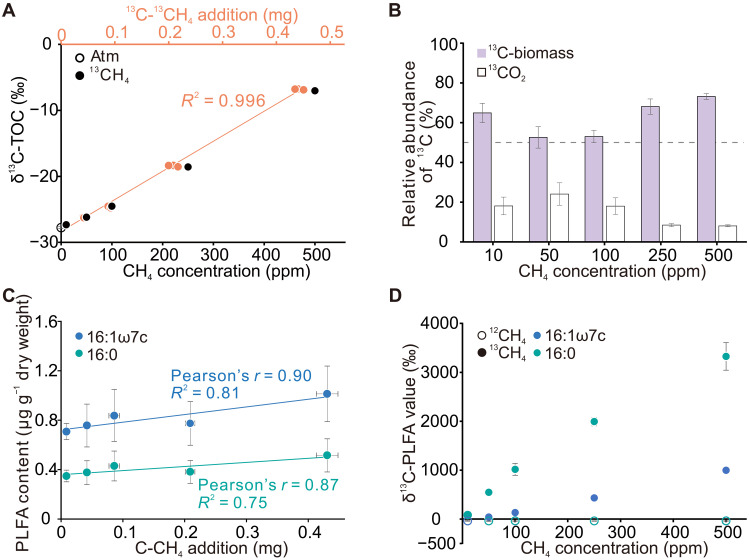
^13^C derived from ^13^CH_4_ incorporation into microbial biomass and carbon assimilation efficiency under different methane concentrations. (**A**) δ^13^C-TOC value after 30 days of incubation plotted against the total amount of ^13^CH_4_ added (orange circle and line) and CH_4_ concentration (black circles). (**B**) Relative abundance of ^13^C incorporated into biomass and CO_2_ from ^13^CH_4_. (**C**) Linear regression of dominant PLFA components (16:0, 16:1ω7c) relative to the total amount of methane added. (**D**) PLFA-specific δ^13^C values (16:0, 16:1ω7c) plotted against ^13^CH_4_ concentrations.

Methane oxidation rates in cave sediments increased markedly with CH_4_ concentration, from 2.9 ± 0.1 ng·g^−1^·hour^−1^ under ambient conditions to 90.7 ± 4.5 ng·g^−1^·hour^−1^ at 500 parts per million (ppm) (table S1). Isotopic labeling experiments showed that both ^12^CH_4_ and ^13^CH_4_ were completely consumed within 3 days following supplementation across the 2 to 500 ppm concentration range (Fig. 1, A and B). The comparable consumption of highly enriched ^13^CH_4_ (99 atom% ^13^C) and ^12^CH_4_ suggested that isotopic enrichment had no detectable effect on the activity of methanotroph (Fig. 1, A and B). After 1 month of incubation with ^13^CH_4_, NanoSIMS imaging revealed significantly increased δ^13^C values in microbial cells exposed to 500 ppm ^13^CH_4_ compared to ^12^CH_4_-treated controls (Fig. 1C), with *P* < 0.001 (Fig. 1D). This cellular-scale increase in δ^13^C was mirrored in the bulk sediment organic carbon. The δ^13^C-TOC increased systematically with rising ^13^CH_4_ concentration, showing a strong linear correlation with methane addition (Fig. 2A). This dose-dependent δ^13^C-TOC shift reflects substantial incorporation of methane-derived carbon into the sediment organic pool. Quantitatively, between 52.6 ± 5.4% and 73.2 ± 1.5% of the supplied ^13^C-^13^CH_4_ was assimilated into microbial biomass, demonstrating efficient methane-derived carbon uptake across all tested concentrations and indicating a substantial flux into microbial organic pools (Fig. 2B). PLFA profiling across CH_4_ treatments consistently revealed that 16:1ω7c and 16:0 were the predominant fatty acids (fig. S1, A to C), indicating relative stable microbial sources contributing to these PLFAs. The relative proportions of 16:1ω7c and 16:0 remained stable, whereas their absolute concentrations increased linearly with methane addition (table S2 and Fig. 2C), indicating biomass accumulation. Since PLFAs degrade rapidly after cell death, the observed δ^13^C enrichment in both 16:0 and 16:1ω7c provides strong evidence for active microbial assimilation of methane-derived carbon, with the enrichment being particularly pronounced in 16:0 (Fig. 2D and fig. S1D). Although many bacteria, including methanotrophs, typically produce these fatty acids, the observed increase in the δ^13^C values of 16:1ω7c and 16:0 with increasing methane supply (Fig. 2C and fig. S1D) strongly indicates that methanotrophs are the primary contributors to these PLFAs in our experiments.

### Low-*K*_m_ USCγ is responsible for methane oxidation in cave sediments

**Fig. 3.**
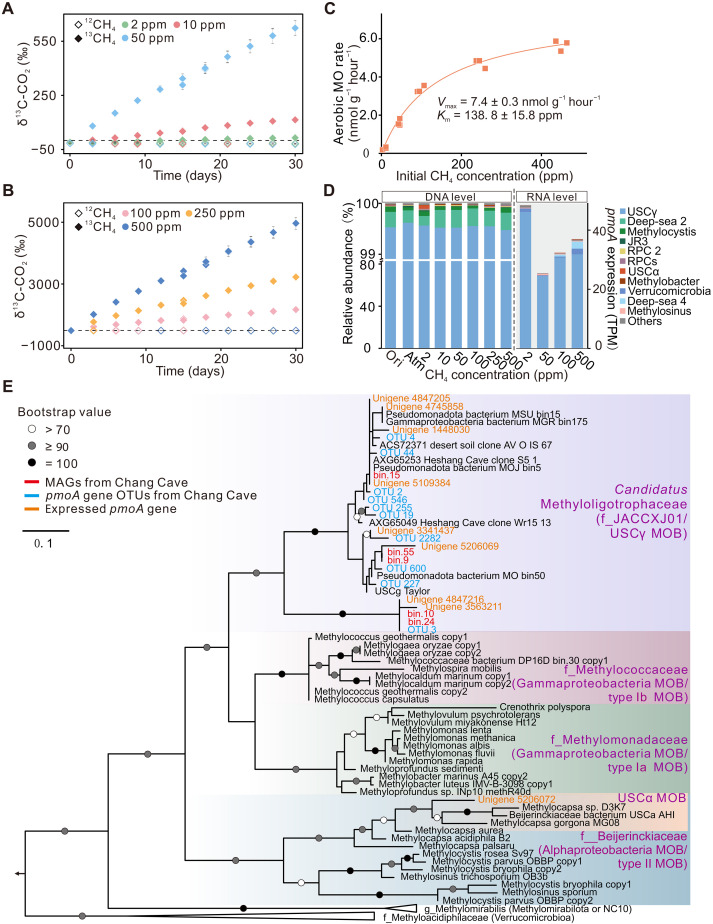
Variations in δ^13^C-CO_2_ values, methane oxidation kinetics, methanotroph community composition, and phylogenetic analysis of *pmoA* in cave sediments. (**A** and **B**) Variations in δ^13^C-CO_2_ values during incubation with ^12^CH_4_ or ^13^CH_4_ at concentrations from 2 to 500 ppm [(A) 2, 10, and 50 ppm; (B) 100, 250, and 500 ppm]. (**C**) Michaelis-Menten kinetics of methane oxidation by atmospheric methanotrophs, showing a *K*_m_ in the micromolar range. (**D**) Left: Methanotrophic community profiling before and after 1-month incubation with varying methane concentrations, based on *pmoA* gene amplicon sequencing (DNA level). Right: Methanotroph community composition based on *pmoA* transcript levels, quantified as transcripts per million. “Ori” refers to original samples, “Atm” denotes samples incubated with ambient air; numbers represent methane concentration (parts per million) used in the incubation. (**E**) The maximum-likelihood phylogenetic tree was constructed based on the amino acid sequences of *pmoA* gene derived from the top 10 most abundant OTUs (blue), MAG-derived low-*K*_m_ methanotroph *pmoA* genes (red), and actively expressed *pmoA* gene (orange). The top 10 OTUs accounted for 58.2% of the total methanotrophic community. Bootstrap values of >70, ≥90, and 100 are represented by white, gray, and black circles, respectively.

Carbon isotope analysis of CO_2_ confirmed sustained methane oxidation activity across all methane concentrations and time points, with δ^13^C-CO_2_ values progressively increasing in response to ^13^CH_4_ addition (Fig. 3, A and B). The *K*_m_ for methane was estimated at 138.8 ± 15.8 ppm (Fig. 3C), which falls within the range of *K*_m_ values observed in pure cultures of USCα (*M. gorgona* MG08) and environmental samples from the upland soils (*16*, *25*).

Amplicon sequencing of the *pmoA* gene across three seasons consistently identified USCγ as the dominant methanotroph within the MOB community, comprising >94% of sequences, with a single seasonal exception observed in winter 2019, when it accounted for 89.3% (fig. S2A). This dominance also persisted in controlled incubations, where USCγ represented up to 99% of the MOB community across all initial CH_4_ concentrations, both pre- and postincubation (Fig. 3D, left). Quantitative polymerase chain reaction (PCR) analysis supported this pattern, with *pmoA* gene copy numbers assigned to USCγ reaching 10^7^ per gram of sediment, approximately three orders of magnitude higher than those assigned to USCα, another clade of atmMOB (fig. S2B). Metatranscriptomic transcript-per-million (TPM) values further confirmed the transcriptional dominance within the methanotrophic fraction (Fig 3D, right). The overall MOB community composition was similar between the original (Ori) sample and the postincubation treatments, as reflected in consistent relative abundance patterns of the top 50 *pmoA* gene operational taxonomic units (OTUs) (fig. S3). By contrast, the three seasonal field samples exhibited substantial differences, both among themselves and compared to the Ori (collected in January 2021) and postincubation samples, likely reflecting seasonal heterogeneity within the cave. However, USCγ remained the dominant methanotroph in all cases. This underscored the ecological resilience and functional stability of the USCγ-dominated methanotrophic community.

### Metagenomics defines the affiliation of USCγ with the family of *Candidatus* Methyloligotrophaceae

**Fig. 4.**
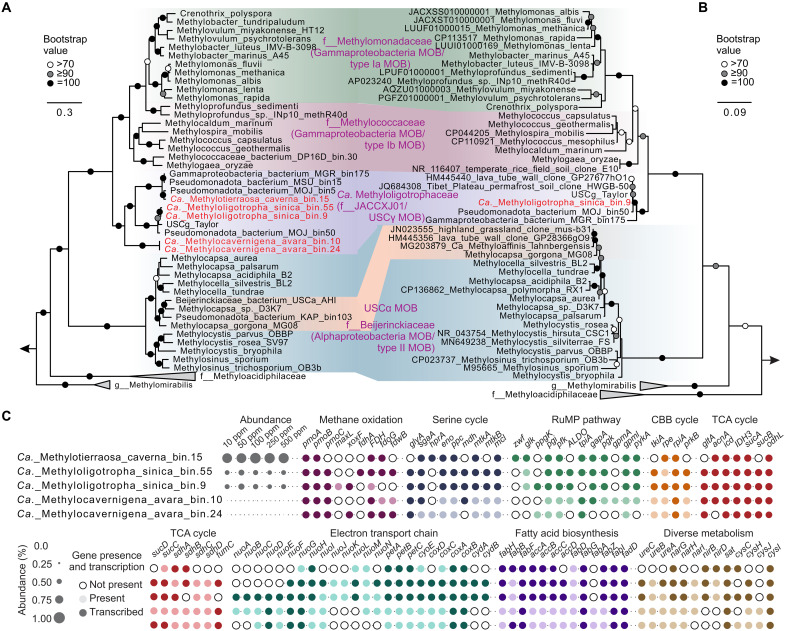
The phylogeny, abundance, and metabolic pathways of methane oxidation and C1 assimilation, as indicated by metagenomic and metatranscriptomic analyses of *Ca.* Methyloligotrophaceae in the sediments of Chang Cave. (**A**) Maximum-likelihood phylogenetic tree based on 120 bacterial single-copy genes from MAGs, illustrating the classification of *Ca.* Methyloligotrophaceae into three genera with two new genera identified. (**B**) Maximum-likelihood phylogenetic tree based on 16*S* rRNA gene sequences recovered from MAG bin9. Bootstrap values are indicated by white, gray, and black circles, representing bootstrap values of >70, ≥90, and 100, respectively. In (A) and (B), genomes recovered from Chang Cave are highlighted in red. (**C**) Relative abundance (percentage) of five representative MAGs inferred from metagenomic read mapping, along with the presence and transcription of their key functional genes. The presence and transcription of genes associated with methane oxidation, the H_4_MPT pathway, the H_4_F pathway, the serine cycle, the RuMP pathway, and the CBB cycle. Circles represent gene presence (gray) or absence (white), with transcription indicated by filled circles.

To resolve the taxonomic identity of the USCγ methanotrophs, we reconstructed five high- or medium-quality MAGs, three of which had >90% completeness (table S3), from CH_4_-incubated cave sediments. Phylogenetic analysis of *pmoA* genes showed that all MAGs clustered within the USCγ clade, forming three well-supported sublineages that corresponded to the top 10 *pmoA* OTUs and the most highly transcribed *pmoA* genes (Fig. 3E). To further resolve their taxonomic affiliations, genome-resolved classification further assigned these MAGs to three distinct species, each affiliated with a separate genus within the newly proposed order *Candidatus* (*Ca.*) Methyloligotrophales, family *Ca.* Methyloligotrophaceae (*26*): *Ca.* Methyloligotropha sinica (bin.55 and bin.9), *Ca.* Methylotierraosa caverna (bin.15), and *Ca.* Methylocavernigena avara (bin.10 and bin.24) (Fig. 4A). Among these, the latter two genera (*Ca.* Methylotierraosa and *Ca.* Methylocavernigena) are newly proposed in this study. This classification was supported by pairwise amino acid identity (AAI) values (table S4), meeting established species- and genus-level thresholds of 95 to 96% and 60 to 85%, respectively (*27*, *28*). Genome-resolved classification and 16*S* rRNA gene phylogenetic analyses, including *Ca.* Methyloligotropha sinica bin.9, consistently corroborate the distinction of this newly defined gammaproteobacterial family, *Ca.* Methyloligotrophaceae, from the canonical MOB families *Methylococcaceae* (type Ib) and *Methylomonadaceae* (type Ia) (Fig. 4, A and B). These MAGs collectively account for ~1.5% of the total microbial community in cave sediments (Fig. 4C). Phylogenetic analysis revealed that all *pmoA* sequences recovered from the metagenome cluster within the family *Ca.* Methyloligotrophaceae, partitioned among *Ca.* Methylotierraosa (51.4%), *Ca.* Methyloligotropha (44.1%), and *Ca.* Methylocavernigena (4.5%) (fig. S4), highlighting their contribution and ecological relevance to trace-methane oxidation in cave ecosystems.

### Flexible methane metabolism and adaptive strategies of *Ca.* Methyloligotrophaceae

Metagenomic and metatranscriptomic analyses revealed that *Ca.* Methyloligotrophaceae harbors a complete and transcriptionally active methane oxidation pathway. The *pmoCAB* gene cluster, encoding particulate methane monooxygenase (pMMO), displayed the highest normalized transcript abundance (Fig. 4C and fig. S5). Transcriptomic mapping to each MAG revealed that *xoxF* (encoding the lanthanum-dependent methanol dehydrogenase) and *fdh*/*fdoG* (encoding the formate dehydrogenase complex) reached up to 8.4, 16.7, and 10.4%, respectively, of the *pmoCAB* gene cluster expression, which was estimated as the mean transcript abundance of the available subunits (*pmoA*, *pmoB*, and *pmoC*) per MAG, supporting a transcriptionally complete methane-to-CO_2_ oxidation pathway (fig. S5). In parallel, genes involved in three potential C1 assimilation pathways were identified and actively transcribed: a near-complete serine cycle (missing the gene encoding glycerate 2-kinase, *gck*), a putative ribulose monophosphate (RuMP) pathway combining a complete Embden-Meyerhof pathway (EMP) and a partial Entner-Doudoroff (EDD) branch, and a partially reconstructed Calvin-Benson-Basham (CBB) cycle (Fig. 4C and fig. S5). However, across the MAG annotations and their corresponding transcriptome mappings, neither genes nor transcripts encoding key enzymes of the RuMP (e.g., hexulose-6-phosphate synthase) and the CBB cycle [e.g., ribulose-1,5-bisphosphate carboxylase/oxygenase (RuBisCO)] were detected, suggesting noncanonical or incomplete forms of these pathways (fig. S5). The MAGs fully encode core metabolic pathways, such as the tricarboxylic acid (TCA) cycle, pentose phosphate pathway, glyoxylate cycle, and methylcitrate cycle. Moreover, these pathways are clearly transcribed (fig. S6A), aligning well with their role in CH_4_-based central metabolism. Cross-mapping of conserved genes at community level may occur and cannot be entirely ruled out.

*Ca.* Methyloligotrophaceae encodes a highly adaptable respiratory system, with actively expressed genes for five terminal oxidase variants and dissimilatory nitrate reduction (e.g., dissimilatory nitrate reductase and nitrite reductase; fig. S6B), which likely facilitate survival across varying, including low, oxygen concentrations (*15*). Moreover, genomes of *Ca.* Methyloligotropha and *Ca.* Methylocavernigena contained and actively expressed genes encoding [NiFe] hydrogenases (fig. S6), which were phylogenetically assigned to group 1 and group 4 based on maximum-likelihood analysis of the amino acid sequences obtained from MAGs (fig. S7), suggesting the potential utilization of H_2_ as an alternative energy source, although this remains to be experimentally confirmed, particularly given the current challenges in obtaining *Ca.* Methyloligotrophaceae in enrichment or pure culture. In addition, the presence and transcription of genes encoding urease and sulfate adenylyltransferase indicate the ability to assimilate nitrogen from urea and sulfur from sulfate (figs. S6B and S8), thereby supporting survival in nutrient-limited cave habitats.

### Transcriptional response to elevated methane concentrations

**Fig. 5.**
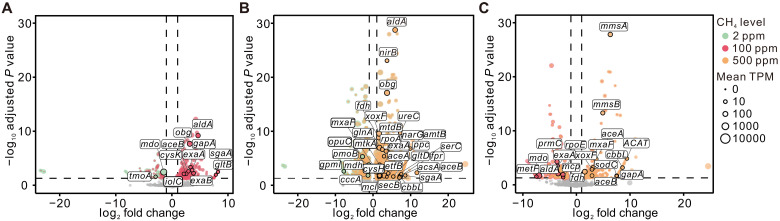
Transcriptional responses of microbial communities in cave sediments to elevated methane concentrations. (**A** to **C**) Volcano plots depicting differential gene expression in cave sediment metatranscriptomes under varying methane treatments: 2 ppm versus 100 ppm (A), 2 ppm versus 500 ppm (B), and 100 ppm versus 500 ppm (C). The horizontal dashed line indicates the significance threshold (adjusted *P* = 0.05), while vertical dashed lines indicate log_2_ fold change cutoffs at −1 and 1. Each point corresponds to a gene, with the size of the point proportional to transcript abundance (TPM). Genes that meet both significance (adjusted *P* < 0.05) and fold-change (|log_2_ fold change| ≥ 1) criteria are highlighted in color; gray points indicate genes that do not meet these criteria. Gene abbreviations and gene products: *tmoA*, toluene monooxygenase; *mdo*, formaldehyde dismutase/methanol dehydrogenase; *comA*, phosphosulfolactate synthase; *obg*, guanosine triphosphatase; *aceB*, malate synthase; *cysk*, cysteine synthase; *iolC*, 5-dehydro-2-deoxygluconokinase; *groEL*, chaperonin GroEL; *aldA*, aldehyde dehydrogenase; *gapA*, glyceraldehyde 3-phosphate dehydrogenase; *exaA*, alcohol dehydrogenase; *exaB*, cytochrome C oxidase; *sgaA*, serine-glyoxylate transaminase; *gltA*, *gltB*, *gltD*, glutamate synthase; *opuC*, osmoprotectant transport system substrate-binding protein; *pmoB*, methane monooxygenase subunit B; *nirB*, nitrite reductase; *xoxF*, lanthanide-dependent methanol dehydrogenase; *fdh*, formate dehydrogenase; *glnA*, glutamine synthetase; *cysH*, phosphoadenosine phosphosulfate reductase; *mxaF*, methanol dehydrogenase; *ureC*, urease; *mtdB*, methylene-H_4_MPT dehydrogenase; *rpoA*, DNA-directed RNA polymerase; *etfB*, electron transfer flavoprotein; *aceA*, isocitrate/methylisocitrate lyase; *narG*, nitrate reductase/ nitrite oxidoreductase; *amtB*, ammonium transporter; *fpr*, ferredoxin/flavodoxin–nicotinamide adenine dinucleotide phosphate reductase; *aceA*, isocitrate/methylisocitrate lyase; *cbbL*, ribulose-bisphosphate carboxylase; *secB*, preprotein translocase; *prmC*, propane 2-monooxygenase; *metF*, methylenetetrahydrofolate reductase; *rpoE*, RNA polymerase sigma-70 factor; *mmsA*, malonate-semialdehyde dehydrogenase; *mmsB*, 3-hydroxyisobutyrate dehydrogenase; *ACAT*, acetyl-CoA C-acetyltransferase.

To investigate transcriptional responses to methane availability, cave sediments were incubated under different methane concentrations (2, 100, and 500 ppm). Transcription profiles between 2 and 100 ppm showed limited overall changes, with significant differences restricted to a small subset of metabolic pathways. At 100 ppm, the genes *mdo* and *tmoA* were repressed, whereas genes involved in carbon assimilation through the serine cycle (*sgaA*), the glyoxylate cycle (*aceB*), and the EMP (*gapA*) were up-regulated. Additional increases were observed in nitrogen metabolism (*gltB*), sulfur metabolism (*cysK*), pyruvate metabolism (*aldA*, *exaA*), electron transfer (*exaB*), and cellular processes (*obg*) (Fig. 5A and fig. S9A). Raising methane to 500 ppm, relative to 2 ppm, resulted in a broad shift in transcriptional profiles. Genes for methane oxidation (except *pmo*, including *xoxF*, *mxaF*, *fdh*) and C1 transfer via the tetrahydromethanopterin (H_4_MPT) pathway (*mtdB*) were significantly up-regulated, together with carbon assimilation through the EMP (*gapA*, *gpml*), the serine cycle (*sgaA*, *ppc*, *mdh*, *mtkA*, *mcl*), the glyoxylate cycle (*aceA*, *aceB*), and the CBB cycle (*cbbL*). Expression also increased for pyruvate metabolism (*aldA*, *exaA*, *acsA*), nitrogen metabolism (*narG*, *nirB*, *glnA*, *ureC*, *amtB*, *gltD*), sulfur metabolism (*cysH*), and electron transfer (*etfB*) (Fig. 5B and fig. S9B). When 500 ppm was compared directly with 100 ppm, significant up-regulations were observed for methane oxidation (*xoxF*, *mxaF*, *fdh*), the glyoxylate cycle (*aceA*, *aceB*), the CBB cycle (*cbbL*), EMP (*gapA*), valine degradation (*mmsA*, *mmsB*), and fatty acid metabolism (*ACAT*) whereas *mdo*, *metF*, *aldA*, *prmC*, and *mcp* were down-regulated (Fig. 5C and fig. S9C). No significant differential expression was detected for *pmo* genes across methane concentrations, which may reflect either a constitutively high baseline expression, as previously reported for *Methylocystis* strains, or a level already sufficient to sustain methane oxidation at higher concentrations (500 ppm) (*29*, *30*). The coordinated up-regulation of downstream oxidation genes (*xoxF*, *mxaF*, *fdh*) therefore suggests that enhanced methane oxidation capacity at 500 ppm is achieved primarily by accelerating methanol and formaldehyde processing rather than by increasing the transcription of *pmo*. Furthermore, MAG-based transcriptome mapping revealed increased expression of oxidative phosphorylation–related genes (*nuoF*, *sucA*, *atpA*, *atpG*, *coxB*) at 500 ppm compared to 2 ppm (table S5). Together, these patterns indicate that while transcriptional changes were limited at 100 ppm, exposure to 500 ppm methane drives a coordinated metabolic response, coupling downstream methane oxidation and carbon assimilation with enhanced nitrogen and sulfur metabolism and energy conservation pathways (Fig. 5 and fig. S9).

### Geographical distribution of *Ca.* Methyloligotrophaceae

To examine the broad ecological relevance of *Ca.* Methyloligotrophaceae, we surveyed their global distribution using global environmental 16*S* rRNA gene datasets. A total of 3841 environmental 16*S* rRNA gene sequences shared >97% identity with two species MAGs of *Ca.* Methyloligotropha, with 3712 assigned to *Ca.* Methyloligotropha sinica bin.9, and 129 to USCg_Taylor, based on Integrated Microbial Next Generation Sequencing (IMNGS) screening (*24*). Among these, 1318 sequences were geospatially resolved with annotated latitude, longitude, and habitat metadata, and clustered with *Ca.* Methyloligotropha sinica bin.9 (Fig. 4B). Despite incomplete metadata in some entries, sequences affiliated with *Ca.* Methyloligotropha were detected across all continents, suggesting a broad global distribution across diverse habitats, including farmland, recognized methane sinks such as forests and grasslands, as well as underrepresented or extreme environments such as caves, hypersaline lakes, polar deserts, and glacial forefields (fig. S10). This broad ecological presence underscores their non-negligible potential role in mitigating methane emissions across diverse terrestrial ecosystems.

### Outstanding methane oxidizing capacity of cave ecosystems

**Fig. 6.**
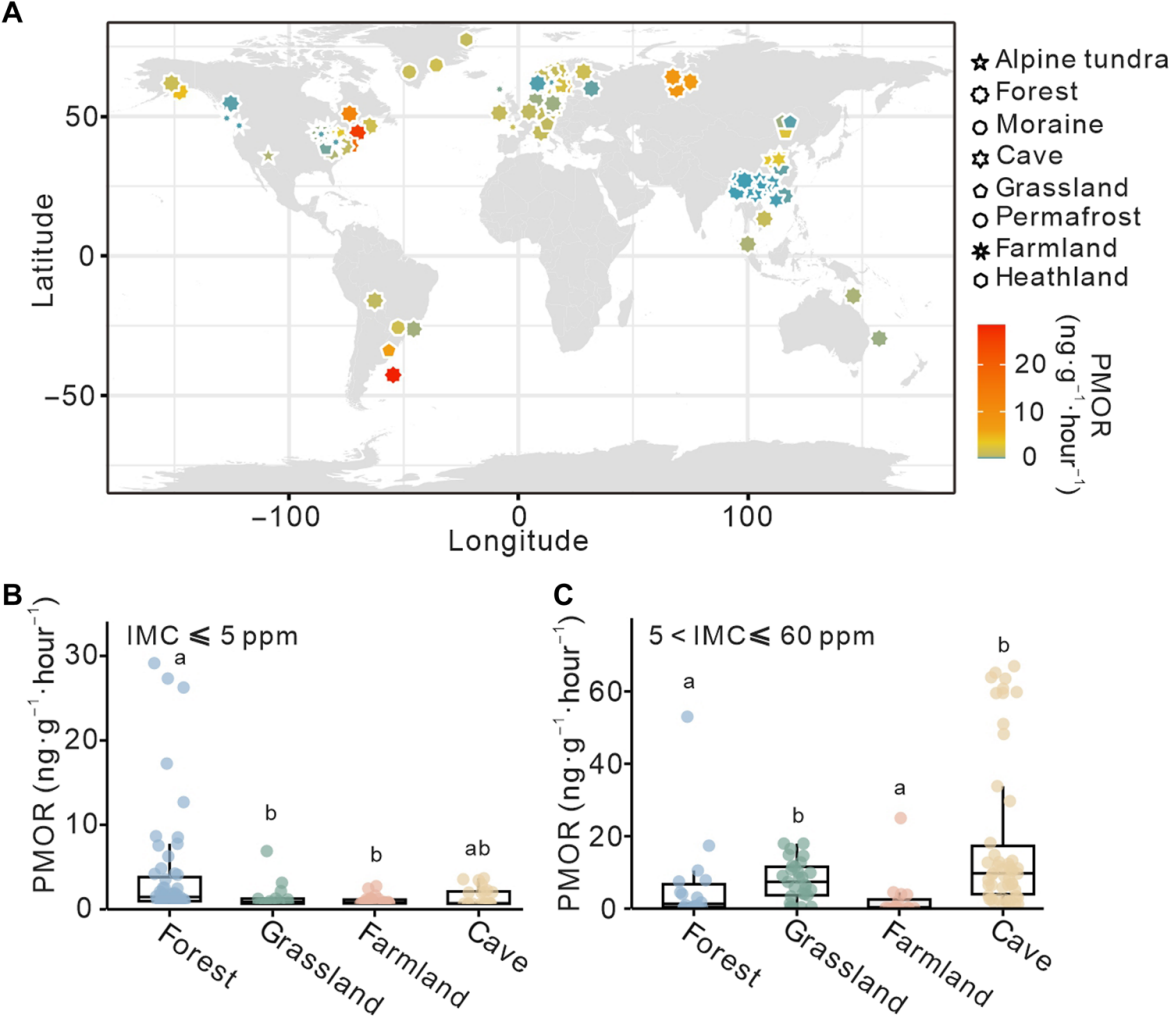
Global distribution and habitat-specific variation of methane oxidation rates. (**A**) CH_4_ oxidation rates in diverse ecosystems worldwide at near-atmospheric methane concentrations. (**B** and **C**) CH_4_ oxidation rates in four representative habitat types (forests, grasslands, farmlands, and caves) are presented to illustrate their capacity of methane oxidation, with CH_4_ concentrations ≤5 ppm (B) and >5 but ≤60 ppm (C). PMOR, potential methane oxidation rates; IMC, initial methane concentration. Letters “a” and “b” denote significant differences in CH_4_ oxidation rates between habitats. Our data for cave habitats are based on original measurement, whereas PMOR data for other habitats were compiled from published studies (the full dataset and references are provided in data S1).

Methane oxidation rates at atmospheric methane concentrations were spatially mapped across terrestrial ecosystems to assess global patterns in relation to habitat type. Habitat-specific differences were observed, with higher rates generally in forests, grasslands, and caves than in farmland and other terrestrial ecosystems, including alpine tundra, heathland, and permafrost (Fig. 6A). Under near-atmospheric conditions (≤5 ppm), cave samples, including sediments and weathered rocks, exhibited high oxidation rates (0.9 ± 1.1 ng CH_4_·g^−1^·hour^−1^), exceeding those in farmlands (0.4 ± 0.5 ng CH_4_·g^−1^·hour^−1^) and grasslands (0.8 ± 1.6 ng CH_4_·g^−1^·hour^−1^), but lower than in forests (3.4 ± 6.5 ng CH_4_·g^−1^·hour^−1^) (Fig. 6B). At elevated methane levels (5 to 60 ppm), oxidation activity increased substantially across all habitats, with caves demonstrating the highest rates (18.8 ± 21.8 ng CH_4_·g^−1^·hour^−1^), surpassing forests (6.3 ± 12.5 ng CH_4_·g^−1^·hour^−1^), grasslands (7.7 ± 5.5 ng CH_4_·g^−1^·hour^−1^), and farmlands (2.6 ± 6.2 ng CH_4_·g^−1^·hour^−1^) (Fig. 6C). These results underscore the substantial but often overlooked role of cave ecosystems as active methane sinks across a wide concentration range.

## DISCUSSION

### Ecophysiological basis for the dominance of *Ca.* Methyloligotrophaceae

The dominance of *Ca.* Methyloligotrophaceae, the lineage previously referred to as USCγ in early *pmoA*-based studies, within MOB community in karst cave sediments is attributed to the confluence of niche conditions favoring this atmMOB, including alkaline pH, low methane concentrations, high Cu bioavailability, and buffered microclimates (*19*, *31*). These conditions define a niche for methanotrophs with low substrate thresholds and adaptive resource allocation strategies, including enhanced stress tolerance and slowed growth rates to reduce biosynthetic costs, and mixotrophic use of alternative substrates under severe energy and carbon limitation (*32*). Methane concentrations used in this study, up to 500 ppm, while above typical atmospheric levels in caves, remained below the ~600 ppm threshold for canonical pMMO induction (*33*) and did not alter the composition of active methanotrophs as inferred from *pmoA* transcripts (Fig. 3D), indicating that conventional methanotrophs were not activated. The isolate culture of atmMOB (*M. gorgona* MG08) can grow under 20% (v/v) CH_4_ (*15*), far above the concentrations used in this study, confirming their high methane tolerance and supporting our incubation design for assessing activity of *Ca.* Methyloligotrophaceae. Kinetic measurements corroborate their low substrate thresholds. The apparent *K*_m_ of *Ca.* Methyloligotrophaceae was 196 nM aqueous CH_4_ (Fig. 3C), within the range reported for upland soils (<200 nM) and closely matching forest soil value (e.g., 161 nM) (*25*, *34*, *35*). Although higher than the low *K*_m_ of 48.5 nM reported for the USCα isolate *M. gorgona* MG08 (*16*), an established atmMOB, it remains well below the micromolar *K*_m_ values commonly observed in conventional methanotrophic isolates (*36*). The specific affinity [*a*^0^_s_ = *V*_max(app)_/*K*_m(app)_] of *Ca.* Methyloligotrophaceae for methane, a metric more appropriate for assessing atmospheric methane utilization efficiency by atmMOB (*37*), was estimated at 0.92 × 10^−9^ liter·cell^−1^·hour^−1^ based on *pmoA* gene quantification (fig. S2B) and a one-gene-per-cell assumption validated in MAGs and the *M. gorgona* MG08 genome. This value is very close to the affinity reported for *M. gorgona* MG08 (1.01 × 10^−9^ liter·cell^−1^·hour^−1^) (*16*), which reinforces the exceptional atmospheric methane-oxidizing capacity of *Ca.* Methyloligotrophaceae.

The lipid biomarker profile of active microbial communities in our samples was characterized by the predominance of PLFAs 16:1ω7c and 16:0. This dominance coupled with their enriched compound-specific δ^13^C signatures (Fig. 2D and fig. S1D) suggested a fundamental contribution of MOB to these PLFAs. However, this interpretation is based on the typical PLFA profiles of conventional MOB (*38*, *39*). PLFA analyses from enrichment cultures or pure isolates of *Ca.* Methyloligotrophaceae would provide solid support for this inference. Phylogenetic analyses based on 16*S* rRNA, *pmoA*, and concatenated metagenomic markers consistently place this lineage within the broader type I methanotroph clade, clustering with *Methylococcaceae* and *Methylomonadaceae* (Figs. 3E and and 4, A and B). Notably, *Ca.* Methyloligotrophaceae exhibits high internal taxonomic diversity and a well-resolved phylogenetic structure, unlike USCα, which is assigned to *Methylocapsa* without strong monophyletic support (*15*).

NanoSIMS imaging and δ^13^C-TOC analyses further revealed that cave atmMOB efficiently assimilate ^13^C-CH_4_ into biomass, with incorporation ranging from 52.6 ± 5.4% to 73.2 ± 1.5% (Figs. 1, C and D, and 2B), demonstrating that atmospheric methane serves as a true source of cellular carbon. Caves are characterized by persistently low methane concentrations and overall oligotrophic conditions, and under such energy-limited environments, efficient use of methane-derived carbon is likely essential for microbial survival. Consistent with this expectation, the assimilation efficiencies observed in cave sediments exceed the 14.3 to 60% range typically reported for paddy soils (*40*, *41*) and approach the 64 to 83% range of landfill soils, where methane-to-oxygen ratios and humidity are regulated under high methane concentrations (>50,000 ppm) (*42*). The efficiencies in caves also surpass the 31 to 54% efficiencies commonly observed for atmospheric methane oxidation in forest soils and tundra soils (*43*, *44*). Despite the increased relative burden of maintenance energy under low substrate availability (*45*, *46*), *Ca.* Methyloligotrophaceae sustain high carbon assimilation efficiency, reflecting metabolic adaptations to persistent carbon limitation. Collectively, these findings underscore the ecological specialization of *Ca.* Methyloligotrophaceae in cave systems and underscore their central role in methane turnover and carbon supply within the subsurface ecosystem.

### Metabolic flexibility underpinning high carbon assimilation efficiency

Although not all the RuMP pathway genes were detected in the MAGs, transcriptomic data showed that the pathway is actively expressed at the community level. Under elevated methane concentrations (500 ppm), genes associated with the EMP pathway, a key branch of the RuMP pathway, were significantly up-regulated (Fig. 5B), suggesting enhanced C1 carbon assimilation. The RuMP pathway, commonly used by type I methanotrophs, is more efficient than the serine cycle used by type II methanotrophs in terms of carbon yield (*47*, *48*). Previous studies have shown that in *Methylococcus capsulatus* and *Methylomicrobium alcaliphilum* 20Z, both phylogenetically affiliated with type I methanotrophs, the EMP and EDD pathways of the RuMP pathway are expressed, with the EMP pathway predominating in C1 carbon assimilation by contributing more significantly to pyruvate, adenosine 5′-triphosphate (ATP), and reduced form of nicotinamide adenine dinucleotide (NADH) production (*49*, *50*). In parallel, genes associated with the serine cycle and serine biosynthesis (Fig. 5B) were also significantly up-regulated, indicating that *Ca.* Methyloligotrophaceae can use this oxygen-insensitive pathway for acetyl–coenzyme A (CoA) synthesis (*51*). The serine cycle enables the synthesis of acetyl-CoA (the C2 building block) from one reduced C1 compound (formate or methanol) and one CO_2_ (generated through formate oxidation) equivalently, without carbon loss (*48*, *51*, *52*). This carbon-conserving feature reflects its hemiautotrophic nature (*53*) and complements the RuMP pathway, particularly under elevated methane conditions (500 ppm) in our experiment. Both C1 assimilation pathways collectively resulted in high carbon conversion efficiency. Genes associated with the CBB cycle were transcribed (figs. S5and S6), and the expression of the key enzyme gene *cbbL* was elevated under 500 ppm CH_4_ compared to 2 ppm (Fig. 5B). However, since *cbbL* was not assembled in the MAGs, it remains uncertain based solely on the transcript sequences whether these atmMOB truly harbor the gene. The CBB cycle has been observed in *M. capsulatus* (type Ib) (*54*), NC10 methanotrophs such as *Ca.* Methylomirabilis oxyfera (*55*), and is also widely present among verrucomicrobial methanotrophs (*56*).

In addition to these C1 pathways, the detection and active transcription of NiFe hydrogenase genes suggests that *Ca.* Methyloligotrophaceae may adopt facultative mixotrophy by using trace H_2_ as an auxiliary energy source to support carbon assimilation under extreme oligotrophic conditions (figs. S6B and S7). The detection of hydrogenase transcripts indicates that trace H_2_ persists in the incubation systems, likely originating from microbial activity or residual atmospheric sources. This leads us to propose the possible presence of low-level H_2_ cycling within oligotrophic caves. Nevertheless, the ecological significance and the underlying microbial processes responsible for H_2_ production remain to be explored. This trait is consistent with Dunfield’s proposition that atmMOB may adopt mixotrophy to survive and mirrors metabolic strategies recently identified in *M. gorgona* MG08 (*16*, *37*, *57*). Previous studies have shown that even non-atmMOB, such as *Methylocystis* and *Methylocapsa*, have mixotrophic metabolism (using CO and H_2_, or CO alone), and such mixotrophic methanotrophs may be capable of oxidizing atmospheric CH_4_ even if they are not strictly classified as atmMOB (*16*). The potential of H_2_ to serve as an alternative energy source is also confirmed in geothermal habitats with abundant H_2_ (>1%, v/v) when the availability of methane fluctuates (*58*). For example, *Methylacidiphilum fumariolicum* SolV can grow autotrophically on H_2_ in the absence of methane (*59*). In addition, *Methylacidiphilum* sp. RTK17.1 exhibits a mixotrophic lifestyle on both H_2_ and CH_4_ (*60*). This metabolic flexibility is hypothesized to facilitate their ecological niche expansion and dominance in variable habitats (*60*). Together, these features reflect a streamlined yet versatile metabolic architecture, enabling *Ca.* *Methyloligotrophaceae* to optimize carbon retention, energy yield, and redox balance across a range of environmental methane availability. Such plasticity likely underpins their ecological success in energy-limited, subsurface ecosystems. Our combined metagenomic and transcriptomic analyses extend the limited insights provided by the sole published MAG (USCg_Taylor), revealing active C1 assimilation, hydrogen (NiFe hydrogenase) utilization, nitrogen and sulfur metabolism, facultative mixotrophy, and phylogenetic subdivision into three distinct genera.

### Ecological significance and climate relevance of *Ca.* Methyloligotrophaceae

Members of *Ca.* Methyloligotrophaceae play a vital role in atmospheric methane oxidation, extending from well-characterized surface ecosystems to the largely unexplored subsurface biosphere. *Ca.* Methyloligotrophaceae, previously identified as the dominant methanotrophs in alpine grassland soils, are primarily responsible for the methane sink function in these ecosystems, in contrast to USCα, which dominate and drive methane oxidation in forest soils (*18*, *20*, *21*). In karst cave systems, environmental conditions such as near-saturated humidity and constant temperatures around the annual mean are generally stable due to the buffering capacity of the subsurface and its delayed response to external climate fluctuations. However, ventilation-driven cave airflow can introduce atmospheric CH_4_ from the surface on seasonal or even daily timescales, depending on cave geometry and external climatic conditions. This process enables periodic gas exchange between the subsurface and the atmosphere (*6*, *7*, *61*). Given their stable microclimatic conditions, minimal exposure to direct human activities, delayed response to environmental perturbations, and relatively undisturbed microbial communities, karst systems may function as more stable and resilient microbial methane sinks than surface soils, which are more vulnerable to disturbance driven by land-use change, agriculture, and climate (*4*, *62*, *63*). This underscores the importance of considering subterranean environments in assessments of the global methane budget.

Although forests and grasslands are well recognized as major terrestrial sinks for methane, covering 40.6 × 10^6^ km^2^ and 5.25 × 10^6^ km^2^ of Earth’s surface, respectively (*64*, *65*), vast expanses of carbonate rock formations, spanning over 20.3 × 10^6^ km^2^ globally (*66*), have remained largely overlooked in this context. Our study shows that subsurface karst environments have a methane oxidation capacity comparable to surface ecosystems (Fig. 6), revealing a substantial yet underappreciated component of the global methane budget.

We estimated the CH_4_ sink in the karst subsurface of southwestern China using two oxidation scenarios (see the Supplementary Materials for detailed parameters), yielding annual sinks of 0.56 and 0.93 Tg CH_4_ year^−1^, respectively. To be conservative, we adopt 0.56 Tg CH_4_ year^−1^ as the representative estimate. This value is comparable to the methane sink capacities of Chinese forests (0.68 Tg CH_4_ year^−1^) and grasslands (0.65 Tg CH_4_ year^−1^) (*67*), offsetting ~5.7% of methane emissions from rice cultivation (9.79 Tg CH_4_ year^−1^) or 2.4% of total agriculture methane emissions in China (23.39 Tg CH_4_ year^−1^) (*68*). Given the limited survey coverage, the heterogeneity of cave morphology, and the spatial variability of methane-oxidizing layers and methanotroph communities, this estimate should be regarded as preliminary. Additional data on methanotroph abundance and diversity, along with further field observations, will be required to refine these calculations before extrapolating beyond southwestern China. These findings underscore a previously unrecognized contribution of subsurface atmMOB to the terrestrial methane budget and point to their potential broader role in natural climate regulation.

Together, our findings establish *Ca.* Methyloligotrophaceae as key agents in methane turnover within karst caves, demonstrating high carbon assimilation efficiency, a low *K*_m_ value, and notable metabolic versatility that reflect their adaptation to energy-limited subterranean environments. Their global distribution across surface and subsurface habitats highlights universal methane-oxidizing capacity, while also revealing subterranean as a critically underestimated methane sink. These insights call for their inclusion in global methane budgets and provide a mechanistic foundation for refining predictions of methane fluxes in terrestrial ecosystems.

## MATERIALS AND METHODS

### Site description and potential methane oxidation rate measurements

Chang Cave, a pristine karst system in Jianshi County, Hubei, China (30°39′26.01″N, 109°58′27.49″E), maintains a stable temperature of 15° to 18°C and near-saturated humidity. The cave contains thick sediment layers and rocks with limited weathering. Sediments were sampled from a site located in the middle section of the cave in July 2019, January 2020, July 2020, and January 2021. Triplicate samples were collected from undisturbed surfaces using sterile spatulas and transported to the laboratory under refrigerated conditions. The samples were either freeze-dried for molecular analyses and stored at −80°C or kept at 4°C for subsequent laboratory incubation. Samples from the first three campaigns were used for MOB community profiling, whereas those from January 2021 were used for methane oxidation rate assays and microcosm incubations. We thank the Chinese authorities (Institute of Karst Geology, Chinese Academy of Geological Sciences, People’s Republic of China) for granting permission to collect karst cave weathered rocks and sediments.

For potential methane oxidation rate measurements, sterile controls were prepared by autoclaving air-dried sediment at 121°C for 20 min on three consecutive days. Ten grams of either sterile or nonsterile sediment was sealed in 250-ml sterile serum bottles, adjusted to 20% moisture content, preincubated overnight at 20°C, and then exposed to varying CH_4_ concentrations (2 to 500 ppm). To ensure the robustness and reliability of the experiments, all treatments were conducted in triplicate. Methane concentrations were monitored using a gas chromatograph (GC) (Agilent 7980, Santa Clara, CA, USA), equipped with a flame ionization detector (FID) and a thermal conductivity cell detector (TCD). A Porapak Q column (80 to 100 mesh, 1.8 m by 3.2 mm by 2.0 mm stainless steel) was maintained at 60°C, with the FID set at 250°C and the TCD set at 220°C, respectively. Nitrogen and helium were used as carrier gases, with a flow rate of 2 ml/min. The methane oxidation capacity of the sediments was determined by monitoring changes in methane concentration over time during active oxidation. The data were fitted to the Michaelis-Menten equation (*69*) using Origin Pro 2022b (OriginLab, Northampton, MA, USA) to obtain precise enzyme kinetic parameters for methane oxidation in the sediments of Chang Cave. Methane concentrations in cave sediment water were calculated using the Bunsen solubility coefficient for CH_4_ at 20°C (0.034) (*70*). Potential methane oxidation rates were also conducted using sediment and weathered rocks from Liangfeng and Dayanqian Caves (Guangxi) under 10 and 50 ppm CH_4_ (*71*).

### Stable isotope microcosm incubations

To investigate methane-derived carbon assimilation, 20 g of air-dried sediment was incubated with either ^13^CH_4_ (99 atom% ^13^C; Reer Technology Co., Ltd., Shanghai, China) or ^12^CH_4_ (unlabeled control) in 125-ml anaerobic vials under aerobic conditions maintained by synthetic air (21% O_2_) for a total of 30 days. Methane concentrations ranged from near-atmospheric (2 ppm) to 500 ppm, deliberately kept below the ~600 ppm threshold for expression and activity of conventional pMMO (*33*), in order to assess methane oxidation potential under moderately elevated CH_4_ conditions relevant to low-*K*_m_ MOB. A killed control consisting of autoclaved sediment was incubated with 2 ppm ^12^CH_4_ to assess nonbiological CH_4_ loss. Methane was replenished every 3 days using synthetic air as the carrier gas, and on day 15, the headspace was replaced with synthetic air prior to continued CH_4_ injection and replenishment. Sediments supplied with ambient air were used to establish natural-abundance δ^13^C baselines, with the headspace completely replaced every 3 days to maintain near-atmospheric CH_4_ concentrations. All experiments were conducted in triplicate at 20°C in the dark.

Headspace gas was sampled before and after each addition to track changes in CH_4_ and CO_2_ concentrations, with δ^13^C-CO_2_ via a GasBench II coupled with a Delta V Advantage continuous flow isotope ratio mass spectrometer (IR-MS; Thermo Fisher Scientific, Bremen, Germany). Ground sediment, passed through a 200-mesh screen, was acidified with 3 M HCl, neutralized, freeze-dried, and analyzed for δ^13^C-TOC using an EA Isolink CN system coupled to a MAT253 Plus mass spectrometer (Thermo Fisher Scientific) (*72*). Carbon utilization efficiency was calculated using Eqs. 1 and 2

$$\text{AT}\left(\%\right)=100\times \frac{\left(\frac{\delta }{1000}+1\right)\times R_{\text{st}}}{1+\left(\frac{\delta }{1000}+1\right)\times R_{\text{st}}}$$ (1)

CSOC13(mg kg−1)=10×TOC(%)×[AT(%)labeled−AT(%)unlabeled]×10 (2)

where AT(%) represents the ^13^C atom abundance; δ is the δ^13^C value; *R*_st_ is the ^13^C/^12^C ratio in the reference standard; ^13^C_SOC_ denotes the assimilated ^13^C content in the sample; TOC(%) is the total organic carbon content; and AT(%)_labeled_ and AT(%)_unlabeled_ refer to ^13^C atom abundance in samples incubated with ^13^CH_4_ and ambient air, respectively.

### NanoSIMS analysis

For cellular-scale isotopic analysis, 3.0 g of sediments incubated with 500 ppm methane was mixed with 5 ml of 0.2% sodium pyrophosphate, and cells were extracted following the method of Liu *et al.* (*73*). Extracted cells were washed twice with phosphate-buffered saline (PBS), fixed in 4% paraformaldehyde (3:1, v/v) at 4°C for 1 to 3 hours, and washed three times by centrifugation (16,000*g*, 5 min). The pellet was resuspended in PBS, mixed with ethanol (1:1), and stored at −20°C.

A 1-μl aliquot of the suspension was applied to conductive silicon wafers, followed by gradient dehydration and critical point drying (*74*). Scanning electron microscopy (Hitachi SU8010, Japan) was operated at an accelerating voltage of 10 to 15 kV to confirm cell presence before NanoSIMS analysis. Isotopic imaging was performed using a NanoSIMS 50L (Cameca, Gennevilliers, France) with a focused Cs^+^ primary ion beam (16 keV) to bombard bacterial cells on gold-sprayed silicon wafers. Secondary ions (^12^C^−^ and ^13^C^−^) were simultaneously collected by multiple electron multipliers. Low-magnification imaging was conducted using a 2-nA primary ion beam for 3 min, scanning areas of 50 μm by 50 μm. High-magnification imaging was performed using a 1.5-pA beam with 2000-μs dwell time and 512 × 512 pixel resolution. Data were processed in WinImage II (version 4.4), analyzing 87 cells incubated with ^12^CH_4_ and 286 with ^13^CH_4_. Statistical differences in isotopic composition were evaluated using Kruskal-Wallis (nonparametric) analysis of variance (ANOVA) in Origin Pro 2022b (OriginLab, Northampton, MA, USA).

### PLFA extraction and analyses

PLFAs were extracted from 5 g of freeze-dried sediment, separated, and converted to fatty acid methyl esters (FAMEs) (*75*, *76*). Compound identification and quantification were performed using an Agilent 7890A GC coupled to an Agilent 5975C mass-selective detector (MS), equipped with a DB-5 MS column (60 m–by–0.25 mm internal diameter, 0.25-μm film thickness) (*77*), referencing published mass spectra (*78*), retention times of FAME standards (Supelco, 47885-U and 47080-U), and identification through MIDI software (MIDI Inc., Newark, DE, USA). In addition, dimethyl disulfide derivatization was used to determine the position of double bonds in monounsaturated fatty acids following the method of Nichols *et al.* (*79*). Compound-specific δ^13^C values were analyzed using a Finnigan Delta XP IR-MS equipped with a DB-5 MS column (60 m–by–0.25 mm internal diameter, 0.25-μm film thickness) (*77*), using squalane (δ^13^C = −19.8‰) as an internal standard, with instrument performance verified before and after each run using FAME standards (Sigma-Aldrich). Duplicate-averaged values (referenced to the Vienna Peedee Belemnite) were corrected for transesterification-derived methyl carbon using the mass balance formula (Eq. 3).

$$\delta^{13}C_{\text{PLFA}}=\left[\left(n+1\right)\times \delta^{13}C_{\text{FAME}}-\delta^{13}C_{\text{methanol}}\right]÷n$$ (3)

where δ^13^C_PLFA_ represents the carbon isotopic value of the fatty acid; *n* is the number of carbon atoms in the fatty acids; δ^13^C_FAME_ and δ^13^C_methanol_ are the measured and known isotopic value of the methylated fatty acid and the methanol used during transesterification, respectively.

### *pmoA* gene sequencing and MAG reconstruction

Both field-collected and subsampled 30-day incubated microcosm samples were freeze-dried immediately after collection. For the incubated microcosms, subsamples were taken directly at the end of incubation, stored at −80°C, and then freeze-dried. Samples for RNA analysis were processed separately as described below. DNA from 0.5 g of freeze-dried sample was extracted using the DNeasy PowerSoil kit (QIAGEN). The *pmoA* gene was amplified with specific primers A189f/A650r (*80*), and sequenced on the Illumina MiSeq platform using PE300 strategy at Shanghai Individual Biotechnology Co., Ltd. (Shanghai, China). Raw data quality control was conducted using Mothur (version 1.39.3) (*81*), and chimeric sequences were removed using VSEARCH (version 2.8.1) (*82*). Amino acid sequences were derived using FrameBot (version 1.1) (*83*) and clustered at 93% sequence similarity using the *uclust* algorithm in USEARCH (version 10.0) (*84*, *85*). Taxonomic assignments were made via BLAST (version 2.10.01) (*86*) against an in-house reference database constructed from published data (*20*, *87*, *88*). Quantitative PCR was performed as previously described (*23*), using primers A189f/gam634r (USCγ) (*89*) and A189f/forest675r (USCα) (*90*) quantified methanotroph abundance on a Bio-Rad CFX96 system (Hercules, CA, USA).

Metagenomic libraries were constructed using the Illumina TruSeq Nano DNA LT Library Preparation Kit and sequenced on the Illumina HiSeq X Ten platform (Illumina, San Diego, CA, USA) using the PE150 strategy at Personal Biotechnology Co., Ltd. (Shanghai, China). Reads were quality-controlled with Cutadapt (version 1.2.1) (*91*) and assembled with MEGAHIT (*92*). MAGs were reconstructed using MetaBat2, MaxBin2, and CONCOCT within the MetaWRAP pipeline (version 1.2.1) (*93*). Only MAGs with >70% completeness and <10% contamination, as evaluated by CheckM (version 1.2.1) (*94*), were retained. Taxonomic assignments were performed using GTDB-Tk (version 2.0.0) (*95*). Phylogenetic tree was constructed with IQ-TREE based on 120 bacterial single-copy genes, aligned using MAFFT (version 7.525) and trimmed with trimAl (version 1.5) using default parameters (*96*–*99*). The 16*S* rRNA gene sequences obtained from the draft genomes using Barrnap (version 0.9; https://github.com/tseemann/barrnap), along with full-length sequences of known methanotrophs from NCBI, were aligned using MAFFT, trimmed with trimAl, and used to construct a phylogenetic tree in IQ-TREE (version 2.2.2.3) with the best-fit model (*96*–*99*). Functional annotation was conducted with DRAM (version 1.3.5), METABOLIC (version 4.0), KEGG BlastKOALA, and eggNOG-mapper (version 2.1.3) (*100*–*103*). AAI was calculated using CompareM (version 0.1.2; https://github.com/dparks1134/CompareM) and EzAAI (version 1.2.3) (*104*), with species- and genus-level thresholds set at 95 to 96% and 60 to 85%, respectively, following established classification standards (*27*, *28*). Additional phylogenetic trees were generated based on the amino acid sequences of pMMO (*pmoA*) and [NiFe]-hydrogenase subunits, aligned using MAFFT and trimmed using trimAl (*96*, *98*, *99*).

### Metatranscriptomic analysis

At the end of the incubation period, RNA analysis samples were briefly supplemented with methane to reach the target concentrations, then immediately collected and flash-frozen in liquid nitrogen to preserve transcriptional signals. Total RNA was extracted using the RNA PowerSoil Total RNA Isolation Kit (12866-25) (MoBio, Carlsbad, CA, USA) and assessed by gel electrophoresis and ultraviolet spectrophotometry. RNA integrity (RIN ≥5.5) was further evaluated using an Agilent 2100 system. After rRNA removal, mRNA was reverse-transcribed, and a macrotranscriptome birdshot sequencing library was constructed using Illumina’s TruSeq Stranded mRNA LT Sample Prep Kit. Sequencing was performed on an Illumina NovaSeq platform with PE150 sequencing strategy at Personal Biotechnology Co., Ltd. (Shanghai, China).

Adapter removal and quality trimming were conducted using Cutadapt (version 1.17) (*91*) and fastp (version 0.20.0) (*105*). rRNA reads were filtered using SortMeRNA (version 4.3.6) (*106*) against a custom database comprising 5SRNAdb and SILVA version 138 (*107*, *108*). De novo assembly was performed with Trinity (version 2.15.1) (*109*), followed by gene prediction using Prodigal and functional annotation with eggNOG-mapper (version 2.1.3) (*102*). Transcript abundances were quantified with RSEM (version 1.3.1) (*110*). Taxonomic annotation of *pmoA* gene sequences was performed via BLAST (version 2.10.01) (*86*) against an in-house reference database. Reads mapping to MAGs was conducted using coverM (*111*), with thresholds of ≥95% nucleotide identity and ≥80% alignment length. Gene counts were exported for downstream analysis. Genes were considered expressed if at least two samples contain 10 or more reads, and TPM values were calculated (*112*).

## Supplementary Materials

### The PDF file includes:

Supplementary Text Figs. S1 to S10 Tables S1 to S5 Legend for data S1 References
