# Supplementary material for: High-efficiency methane consumption by atmospheric methanotrophs in subsurface karst caves: The irrefutable methane sink
Source: Sci Adv. 2026 Feb 4;12(6):eady5942. doi: 10.1126/sciadv.ady5942 (PMC12871462; doi:10.1126/sciadv.ady5942)
Supplement: Supplementary file 1 — Supplementary Text Figs. S1 to S10 Tables S1 to S5 Legend for data S1 References [file sciadv.ady5942_sm.pdf]

Science Advances
AAAS

Supplementary Materials for

High-efficiency methane consumption by atmospheric methanotrophs in subsurface karst caves: The irrefutable methane sink

Xiaoyan Liu *et al.*

Corresponding author: Hongmei Wang, hmwang@cug.edu.cn or wanghmei04@163.com

Sci. Adv. **12**, eady5942 (2026)
DOI: 10.1126/sciadv.ady5942

The PDF file includes:

Supplementary Text
Figs. S1 to S10
Tables S1 to S5
Legend for data S1
References

Other Supplementary Material for this manuscript includes the following:

Data S1

Supplementary Text

Global distribution of *Candidatus* Methyloligotrophaceae and compilation of methane oxidation rates across major methane sinks

To further investigate the global distribution of *Ca*. Methyloligotrophaceae, the 16S rRNA sequences from the *Ca*. Methyloligotropha sinica bin.9 (827 bp, recovered in this study) and the previously published draft genome sequence USCg_Taylor (GCA_002007425.1, 424 bp) (*24*) were used to query Short Read Archive (SRA) datasets via IMNGS (*114*), applying a 97% nucleotide sequence identity threshold and a minimum sequence length of 200 nucleotides. Short sequences obtained via IMNGS were aligned with SINA (*115*), and phylogenetic placement within 16S rRNA tree was performed using the Evolutionary Placement Algorithm (EPA) implemented in RAxML (*116*). Protocols for read retention and acquisition of geographic coordinates (latitude and longitude) followed previously published methods (*15*). The coordinates of positive read sets were visualised using the R packages "ggplot2" and "rnaturalearth".

The methane oxidation rate data were compiled through a comprehensive search of published literature using Google Scholar and the Web of Science, with keywords including "methane (CH_4) oxidation rate", "methane (CH_4) oxidation potential", and "methane (CH_4) oxidation capacity". Relevant studies were selected based on their reporting of methane oxidation rates across various ecosystems. Where available, methane oxidation rates were directly extracted from the text or digitized from figures using the Origin Pro 2022b. Geographical coordinates (latitude and longitude) and experimental methane concentrations were also recorded. Collected data and corresponding references are provided in Data S1. Global visualization of the compiled data was conducted using R packages "ggplot2" and "ggstar". Boxplots were generated to illustrate the distribution of methane oxidation rates across habitats, and statistical differences among habitats were assessed using the Kruskal-Wallis test (nonparametric ANOVA) in Origin Pro 2022b.

Estimation of methane consumption by subsurface atmospheric methane-oxidizing bacteria (atmMOB)

To estimate the potential capacity for atmospheric methane oxidation by atmMOB in karst subsurface environments, we used a conservative, geometry-based calculation that integrates cave dimensions, porosity, and measured oxidation rates. Chang Cave was idealized as a half-cylindrical conduit with a 5 m radius and 500 m length, representing a relatively large cave within the morphologically and volumetrically diverse karst caves of southwestern China, whose low surface-area-to-volume ratios yield conservative estimates of CH_4 oxidation potential. Methane consumption was calculated as the sum of contributions at the sediment–air and weathered rock-air interface, assuming effective oxidation depths of 3 cm and 2 cm, respectively, and a solid bulk density of 1.1 $g{\cdot}cm^{-3}$.

We used 0.9 $ng{\cdot}g^{-1}{\cdot}h^{-1}$, the arithmetic mean (0.9 $ng{\cdot}g^{-1}{\cdot}h^{-1}$) of compiled methane oxidation rates measured on cave samples at near-atmospheric CH_4 (Fig. 6B), as the low oxidation rate. The maximum measured oxidation rate of 3.0 $ng{\cdot}g^{-1}{\cdot}h^{-1}$ in Chang Cave sediments at 2 ppm CH_4 was incorporated into our calculations as the high oxidation rate. When maximum CH_4 oxidation rates measured in weathered rocks from Liangfeng and Dayanqian Caves (Guangxi) were scaled to 2 ppm using the Michaelis–Menten relation, they converged with this value. The oxidation rate was scaled downward with the Michaelis–Menten relation formula (4) (*69*).

$$v = V_{max} \frac{[S]}{K_m + [S]} \quad (4)$$

where v is the CH_4 oxidation rate, v_{max} is the maximum CH_4 oxidation rate, $[S]$ is the methane concentration, and K_m is the half-saturation constant determined in this study. Even when applying the high oxidation rate within a 3 cm sediment–air effective depth, the resulting methane consumption flux remains slightly lower on a per-area basis (1.72 $nmol{\cdot}s^{-1}{\cdot}m^{-2}$) than field-based depletion estimates (1.98 $nmol{\cdot}s^{-1}{\cdot}m^{-2}$) derived by expanding cave surface area from 280 to 1,000 m^2 (*113*), underscoring the conservative nature of the estimation.

We assumed gas exchange between atmospheric air and subsurface air within the upper 40 m of weathered limestone, which has an average porosity of approximately 20% (v/v) (*13, 117*). Chang Cave was used as the reference system for flux calculations, and regional scaling was performed by expressing the subsurface volume of the southwestern China karst region as the equivalent number of Chang Cave–sized volumes. Maximum methane oxidation was taken to occur during periods of active cave–atmosphere ventilation, triggered when the temperature difference (ΔT) between cave air and the external atmosphere exceeds 5.5°C (*113*). Based on this framework, we evaluated two scenarios for estimating the annual CH_4 sink. In the first scenario, the low oxidation rate (0.9 $ng \cdot g^{-1} \cdot h^{-1}$) was applied throughout the year, providing a conservative estimate. In the second scenario, we incorporated seasonal ventilation patterns observed in Shawan Cave, located in southwestern China, where $\Delta T > 5.5$°C persisted for roughly half of the year (*113*). Under this approach, the annual CH_4 sink was estimated by applying the high oxidation rate during the half-year period of active ventilation.

Etymological information

All assembled genomes belong to the family *Ca*. Methyloligotrophaceae of the order *Ca*. Methyloligotrophales as previously defined (*26*). Based on high- and medium-quality metagenome-assembled genomes (MAGs) recovered from Chang Cave sediments, we propose two new genera and one new species within *Ca.* Methyloligotropha.

Species:

'*Candidatus* Methyloligotropha sinica' (si'ni.ca. L. fem. adj. *sinica*, Chinese; N.L. fem. adj. *sinica*, of the cave sediments in China where the bacterium was identified)

'*Candidatus* Methylotierraosa caverna' (ca.ver'na. L. fem. n. *caverna*, cave; N.L. fem. n. *caverna*, of the cave environment from which the bacterium was recovered)

'*Candidatus* Methylocavernigena avara' (a.va'ra. L. fem. adj. *avara*, greedy; N.L. fem. adj. *avara*, associated with environments of low methane availability where the bacterium was

detected)

Genus:

'*Candidatus* Methylotierraosa' (Meth.y.lo.ti.er.ra.o'sa. N.L. neut. n. *methylum*, from French *méthyle*, back-formation from *méthylène*; L. fem. n. *terra*, land; L. fem. adj. -*osa*, full of / abundant in; N.L. fem. n. *Methylotierraosa*, a methyl- using bacterium associated with terrestrial environments)

'*Candidatus* Methylocavernigena' (Meth.y.lo.ca.ver.ni.ge'na. N.L. neut. n. *methylum*, methyl group; L. fem. n. *caverna*, cave; L. fem. adj. -*gena*, originating from; N.L. fem. n. *Methylocavernigena*, a methyl-oxidizing bacterium originating from caves)

Full Classification:

- Domain: Bacteria
- Phylum: Pseudomonadota
- Class: Gammaproteobacteria
- Order: *Candidatus* Methyloligotrophales
- Family: *Candidatus* Methyloligotrophaceae
- Genera and species:
    - Genus: *Candidatus* Methyloligotropha
        - Species: *Candidatus* Methyloligotropha sinica
    - Genus: *Candidatus* Methylotierraosa
        - Species: *Candidatus* Methylotierraosa caverna
    - Genus: *Candidatus* Methylocavernigena
        - Species: *Candidatus* Methylocavernigena avara

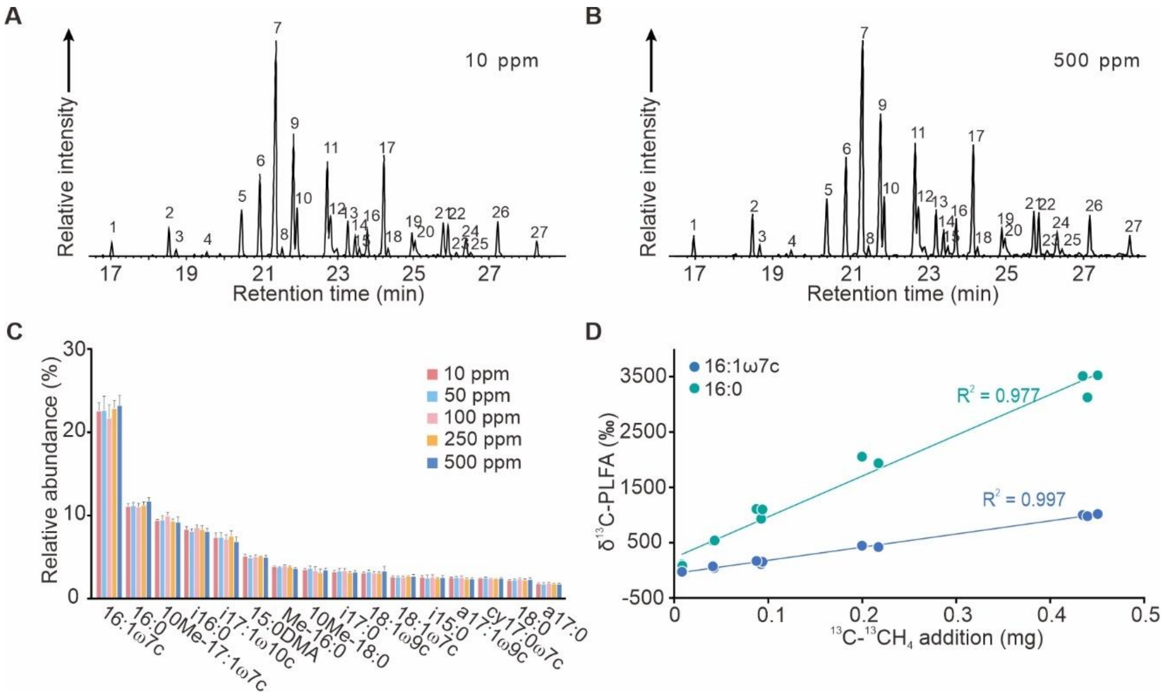

Fig. S1. Total composition and relative abundance of phospholipid fatty acids (PLFAs), and compound-specific δ^{13}C-PLFA values under different methane concentrations.

The total ion chromatogram of PLFAs after 30 days' incubation under 10 ppm $^{12}CH_4$ (**A**) and 500 ppm $^{12}CH_4$ (**B**), with peak assignments as follows: 1. 14:0; 2. *i*15:0; 3. *a*15:0; 4. 15:0; 5. 15:0DMA; 6. *i*16:0; 7. 16:1ω7c; 8. 16:1ω5c; 9. 16:0; 10. Me-16:0; 11. *i*17:1ω10c; 12. *a*17:1ω9c; 13. *i*17:0; 14. *a*17:0; 15. 17:1ω8c; 16. *cy*17:0ω7c; 17. 10Me-17:1ω7c; 18. 17:0; 19, Me-17:1; 20. 10Me-17:0; 21. 18:1ω9c; 22. 18:1ω7c; 23. 18:1ω5c; 24. 18:0; 25. 10Me-18:1ω7c; 26. 10Me18:0; 27. *cy*19:0ω7c. Here, *i*/*a* indicates iso/anteiso alkyl chain; the number preceding Me indicates the methyl group position counting from the carboxyl group; the number preceding a colon indicates the total carbon number; the number after the colon indicates the number of double bonds; the number following ω indicates the position of unsaturation bond, counted from the terminal methyl group; and c indicates a cis geometric isomer. **C**, The main PLFA distribution profiles showing the relative abundance of the top 16 PLFAs after 30 days' incubation with different concentrations of CH_4. **D**, The linear regression analysis between the individual δ^{13}C value of C16:0 and C16:1ω7c and the total ^{13}C-$^{13}CH_4$ addition during the experiments, respectively.

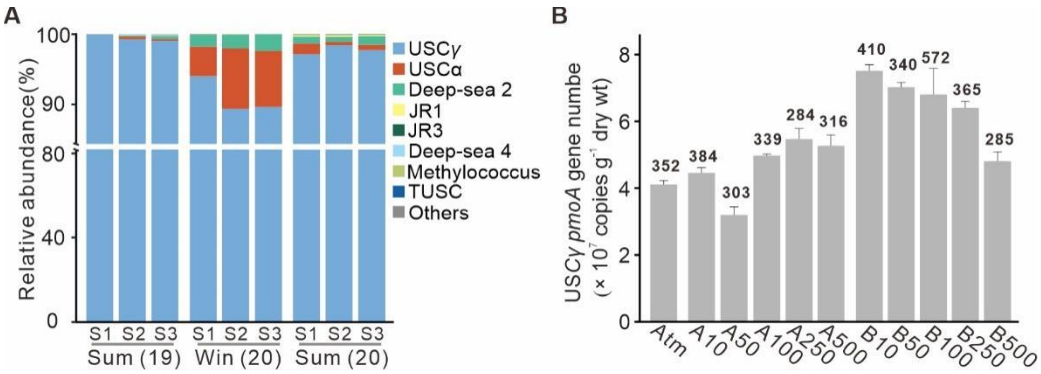

Fig. S2.

Methanotroph community profiling and quantification of Upland Soil Cluster (USC) group in cave sediments based on *pmoA* gene.

A, Methanotroph community profiling as revealed by high throughput sequencing of *pmoA* gene in cave sediments collected during summer 2019, winter 2020, and summer 2020. **B**, Quantification of USC gamma (USCγ) and USC alpha (USCα) after one-month incubation with different methane concentrations. Bold numbers above the bars denote the ratio of USCγ to USCα abundance. Atm: the control group under ambient atmosphere. Groups labeled 'A' received the addition of $^{12}CH_4$, whereas groups labeled 'B' received the addition of $^{13}CH_4$. The concentrations of methane during incubation are represented by 10, 50, 100, 250, and 500 for 10 ppm, 50 ppm, 100 ppm, 250 ppm, and 500 ppm, respectively.

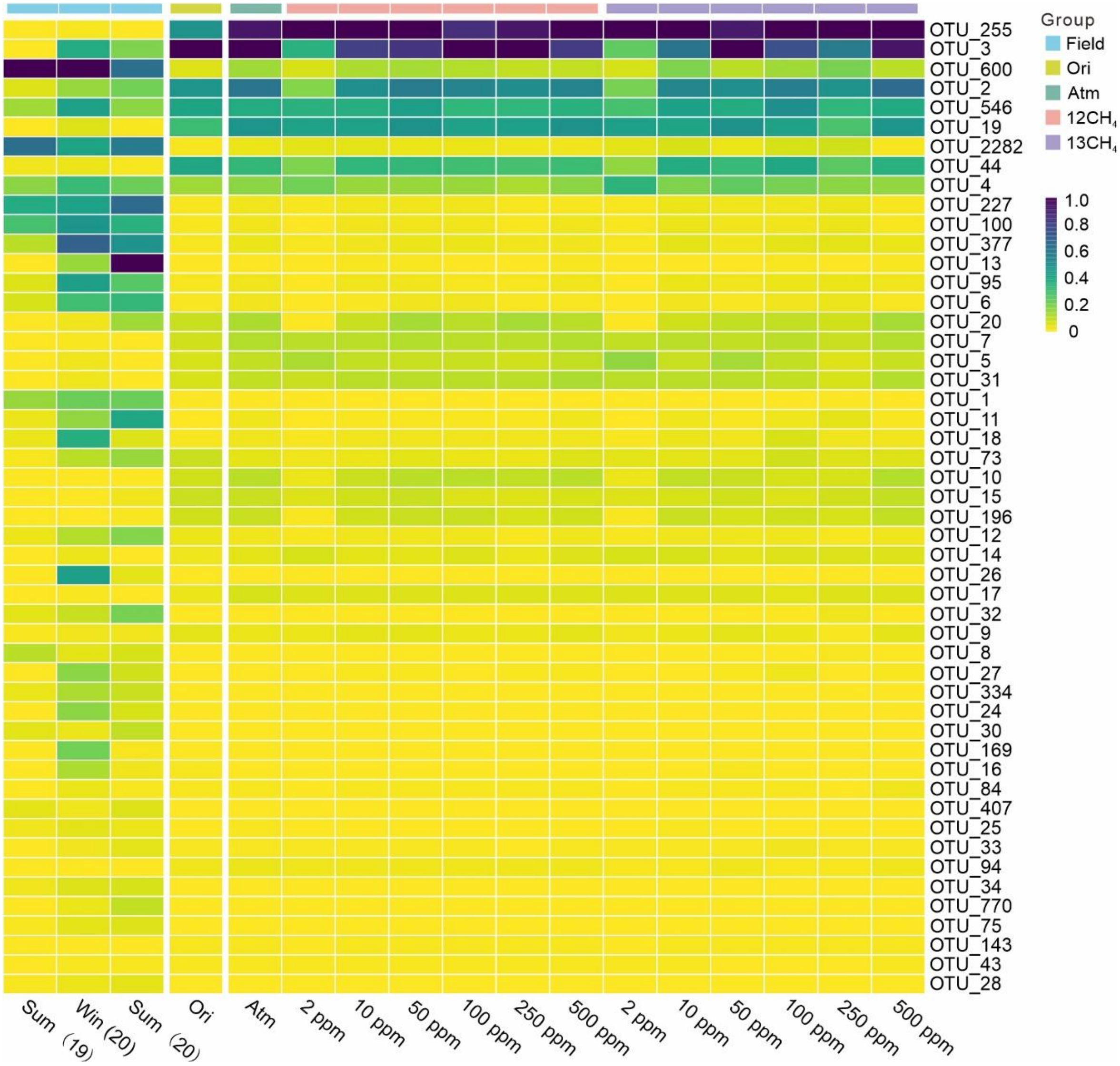

Fig. S3.

Heatmap of top 50 *pmoA* OTUs in the sediments collected from Chang Cave.

The heatmap illustrates the relative abundance distribution of the top 50 OTUs in Chang Cave sediments, as determined by *pmoA* gene amplicon sequencing. These OTUs collectively account for approximately 80.2% to 91.6% of the total methanotrophic communities. Samples include field-collected cave sediments from summer 2019, winter 2020, and summer 2020 (Field); sediments collected in winter 2021 prior to laboratory incubation (Ori); and sediments incubated for 30 days under atmospheric conditions (Atm), with $^{12}CH_4$ (2, 10, 50, 100, 250, and 500 ppm) or with $^{13}CH_4$ (2, 10, 50, 100, 250, and 500 ppm).

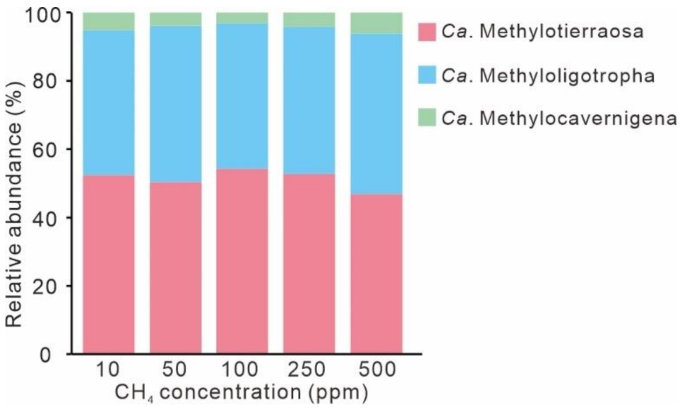

Fig. S4. Composition of methane-oxidizing bacterial communities.

Composition of methane-oxidizing bacterial communities based on *pmoA* gene sequences obtained by metagenomic analysis of Chang Cave sediments incubated for 30 days under CH_4 concentrations of 10, 50, 100, 250, and 500 ppm.

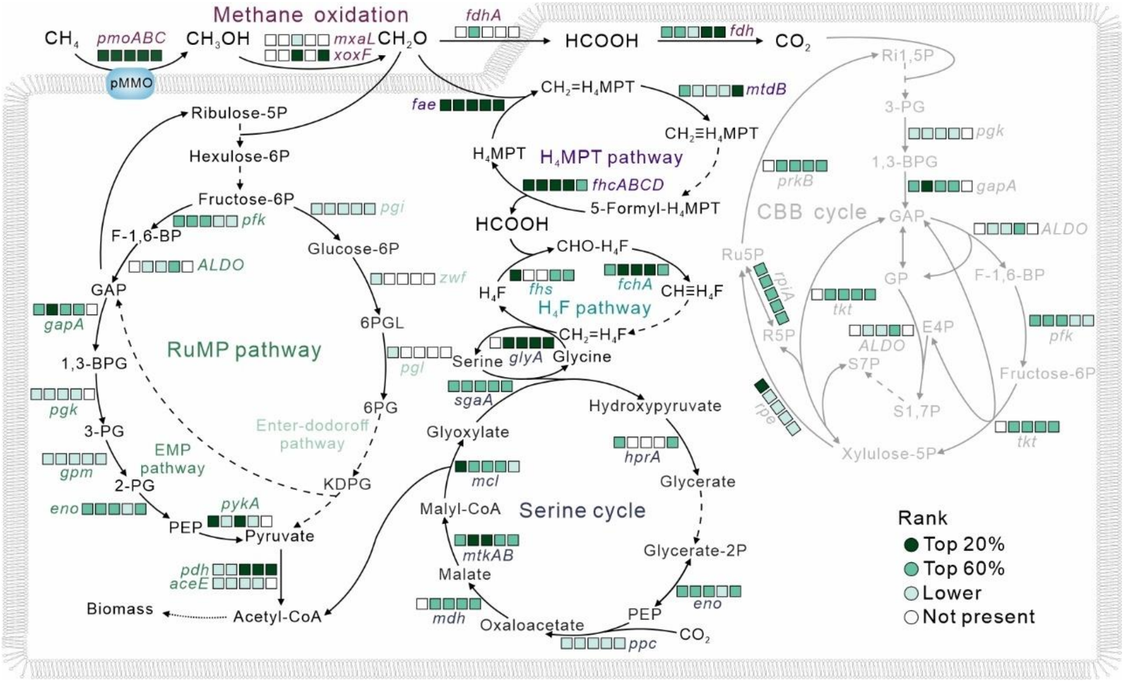

Fig. S5.

Potential metabolic pathways for methane oxidation and C1 assimilation, as indicated by the metagenome assembly of *Candidatus* Methyloligotrophaceae.

The squares, arranged from left to right, represent different bins (e.g., bin.15, bin.55, bin.9, bin.10, bin.24). Solid lines (black or gray) denote genes that are present and transcribed, whereas dashed lines (black or gray) indicate genes that were not annotated in the metagenome-assembled genomes (MAGs). Pathways, intermediates, and genes with uncertain presence are shown in gray to reflect this uncertainty. Gene transcript abundance, normalized to Transcripts Per Million (TPM), was represented by colour intensity, with darker colours indicating higher abundance and white indicating gene absence. 6PGL: Glucono-1,5-lactone-6-phosphate; 6PG: 6-Phosphogluconate; KDPG: 2-Dehydro-3-deoxy-6-phospho-D-gluconate; F-1,6-BP: Fructose 1,6-bisphosphate; GAP: Glyceraldehyde-3-phosphate; 1,3-BPG: Glyceraldehyde-1,3-phosphate; 3-PG, Glycerate-3-phosphate; 2-PG: Glycerate-2-phosphate; PEP: Phosphoenolpyruvate; Ru5P: Ribulose-5-phosphate; Ri1,5P: Ribulose-1,5-bisphosphate; R5P, Ribose-5-phosphate; E4P: Erythrose-4-phosphate; GP: Glycerone phosphate; S1,7P: Sedoheptulose-1,7-bisphosphate; S7P: Sedoheptulose-7-phosphate.

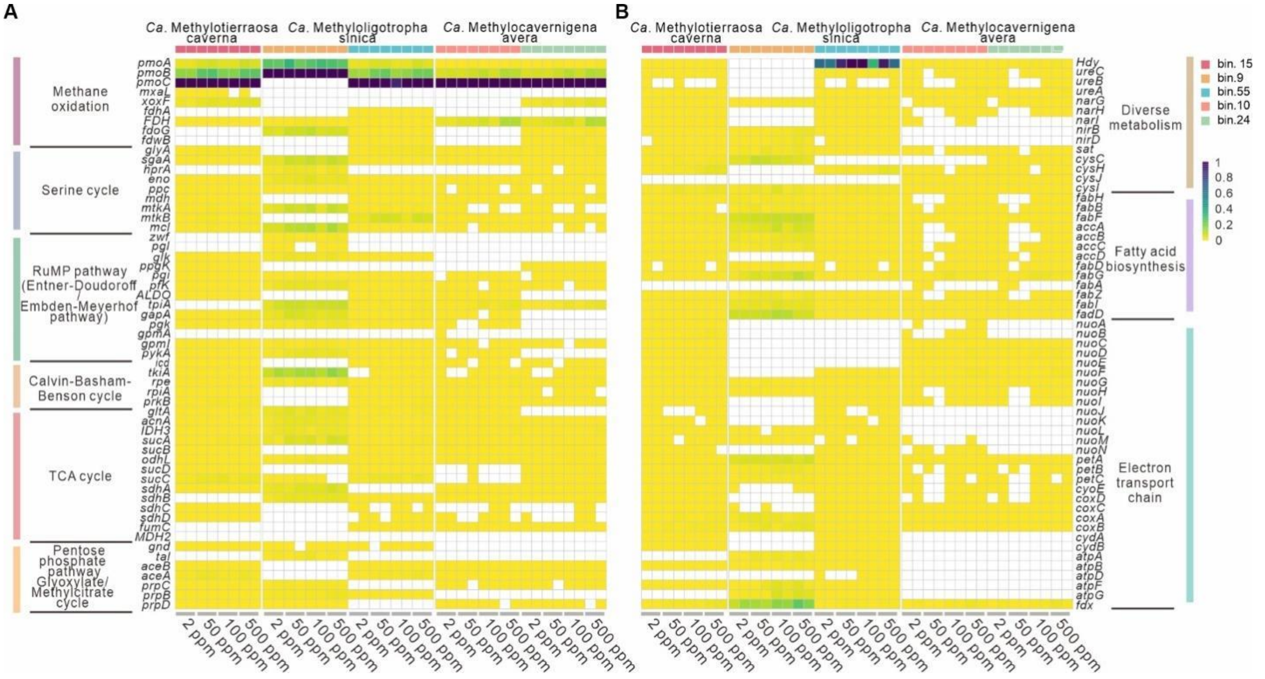

Fig. S6.

Transcriptomic profiling reveals distinct differences in metabolic pathway utilization among the three genera within *Ca.* Methyloligotrophaceae in sediments from Chang Cave.

The heatmap illustrates the relative gene expression profiles in *Ca.* Methyloligotrophaceae MAGs related to key metabolic pathways, including methane oxidation, the serine cycle, the ribulose monophosphate (RuMP) pathway (comprising both the Entner-Doudoroff and Embden-Meyerhof pathways), the Calvin-Basham-Benson (CBB) cycle, the tricarboxylic acid (TCA) cycle, the pentose phosphate pathway, the glyoxylate cycle, and the methylcitrate cycle, as shown in panel (**A**). Panel (**B**) presents transcript abundance for genes involved in various additional metabolic processes, including hydrogen, urea, nitrogen and sulfur metabolism, as well as components of the electron transport chain and fatty acid biosynthesis. Gene expression levels are quantified as normalized transcripts per million across different methane concentrations, with each condition represented by two biological replicates. Heatmap colour gradients range from yellow (low expression) to dark blue (high expression), while white indicates either unannotated genes or genes with zero expression.

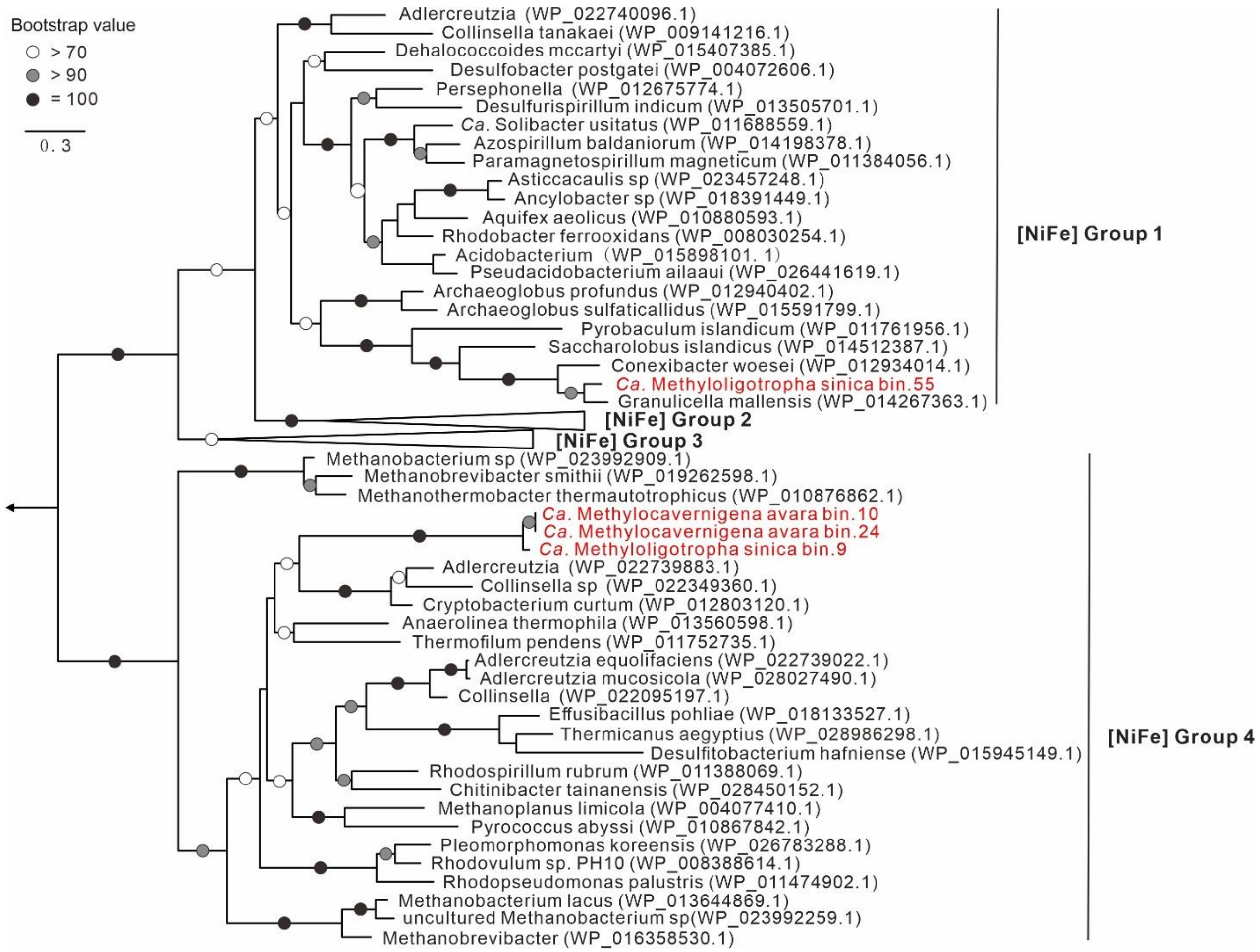

Fig. S7.

The Maximum-Likelihood phylogenetic tree of H_2 hydrogenase amino acid sequences from *Ca.* Methyloligotrophaceae.

The phylogenetic tree was constructed using the Maximum-Likelihood method to analyze the amino acid sequences of H_2 hydrogenase obtained from MAGs of *Ca.* Methyloligotrophaceae. Bootstrap values, calculated from 1,000 resamplings, are indicated at branch points by circle symbols: white (⩾70), gray (⩾90), and black (100).

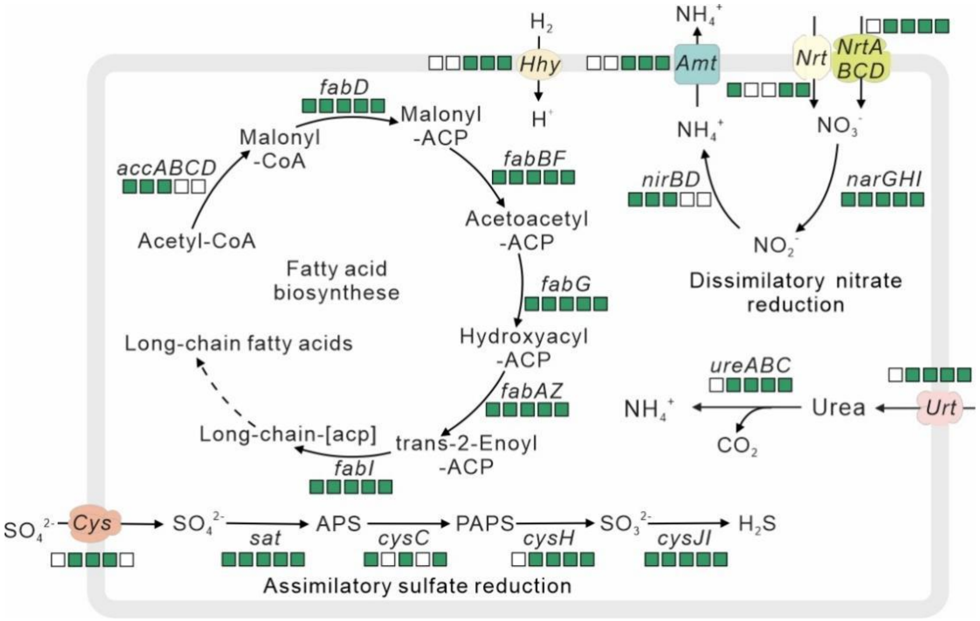

Fig. S8.

Potential metabolic pathways for fatty acid synthesis and diverse metabolic functions inferred from the metagenome assembly of *Ca*. Methyloligotrophaceae.

The squares, arranged from left to right, represent different bins (e.g., bin.15, bin.55, bin.9, bin.10, bin.24), with pathways for fatty acid biosynthesis, dissimilatory nitrate reduction, urease activity, assimilatory sulfate reduction, and hydrogen metabolism. Solid lines show present genes, while dashed lines show genes not annotated in the assembled MAGs.

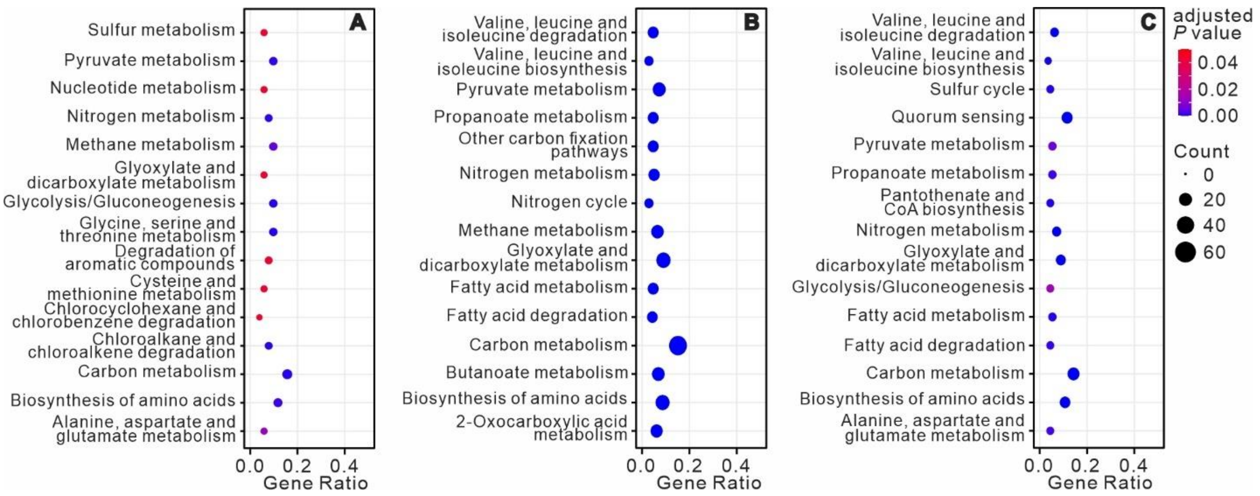

Fig. S9.

Functional profiles of microbiota in cave sediment under different methane concentrations.

Kyoto Encyclopedia of Genes and Genomes (KEGG) pathways enriched among differentially expressed genes for 2 vs. 100 ppm (**A**), 2 vs. 500 ppm (**B**), and 100 vs. 500 ppm (**C**). Differentially expressed genes were identified using DESeq2 with $|\log_2 \text{fold change}| > 1$ and adjusted P value < 0.05. Dot size represents the number of KEGG Orthologs annotated to each KEGG pathway. P values were calculated using hypergeometric tests and adjusted for multiple testing by the Benjamini–Hochberg method. All pathways shown are significant (adjusted $P < 0.05$), and dot colour reflects the adjusted P value, with smaller P values indicated by a shift toward blue.

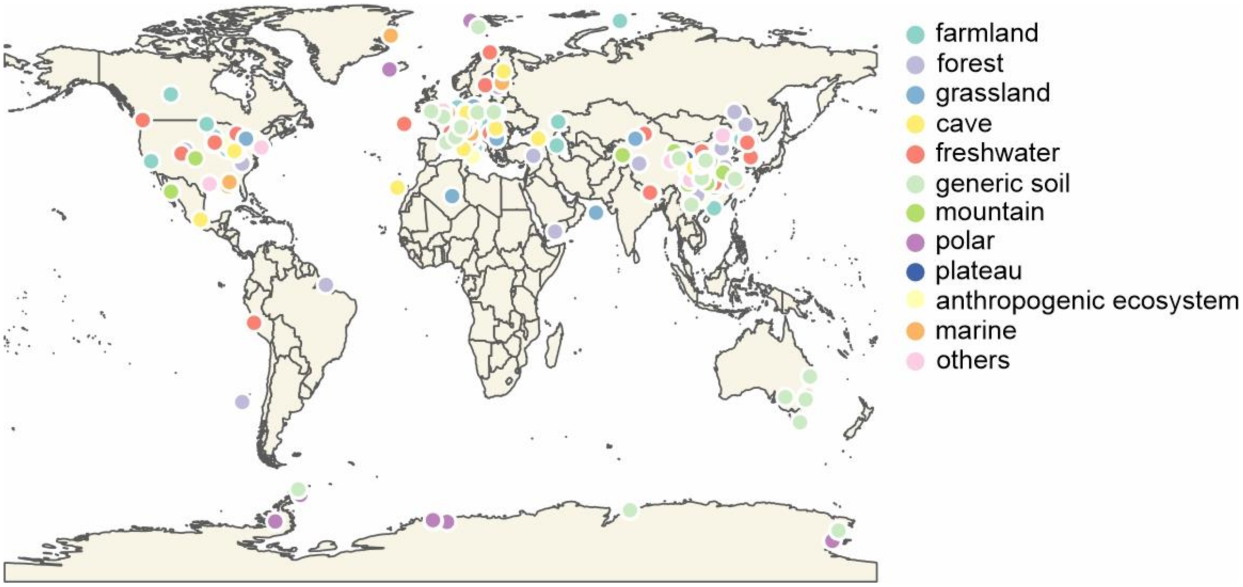

Fig. S10.

Global distribution of *Ca*. Methyloligotrophaceae and their habitats.

The map displays the geographic distribution of *Ca*. Methyloligotrophaceae based on the publicly available Short Read Archive datasets containing environmental 16S rRNA sequences. A total of 1,318 sequences with significant similarity (>97%) to the 16S rRNA of *Ca*. Methyloligotropha sinica bin.9 were identified and projected on the world map based on their valid geographic metadata.

Table S1. Methane oxidation activity of Chang Cave sediments.

The methane oxidation rates of cave sediments collected from the Chang Cave under different CH_4 concentrations.

CH_4 concentration added in microcosm (ppm)	CH_4 oxidation rates (ng $CH_4 \cdot g^{-1} \cdot h^{-1}$)
2	2.9 ± 0.1
10	4.7 ± 0.5
50	25.8 ± 2.9
100	53.5 ± 2.9
250	75.4 ± 3.7
500	90.7 ± 4.5

Table S2. Relationship between phospholipid fatty acids content and methane input.

The linear regression analysis between the absolute content of PLFAs and the total methane addition during the microcosm incubation experiments.

PLFA identified	Pearson's *r*	adjusted R^2	intercept	slope
14:00	0.52	0.23	0.04	0.05
i15:0	0.34	0.07	0.08	0.06
a15:0	0.33	0.06	0.02	0.02
15:00	0.34	0.07	0.01	0.01
15:0DMA	0.37	0.09	0.17	0.11
i16:0	0.33	0.06	0.28	0.17
**16:1ω7c**	**0.53**	**0.24**	**0.72**	**0.61**
16:1ω5c	0.41	0.13	0.02	0.01
**16:00**	**0.5**	**0.21**	**0.36**	**0.33**
Me-16:0	0.28	0.03	0.13	0.06
i17:1ω10c	0.33	0.06	0.24	0.11
a17:1ω9c	0.29	0.03	0.08	0.04
i17:0	0.29	0.03	0.11	0.06
a17:0	0.3	0.04	0.06	0.03
17:1ω8c	0.32	0.06	0.02	0.01
cy17:0ω7c	0.38	0.1	0.08	0.05
10Me-17:1ω7c	0.3	0.04	0.32	0.17
17:00	0.28	0.03	0.02	0.01
Me-17:1	0.35	0.07	0.06	0.04
10Me-17:0	0.23	0	0.03	0.01
18:1ω9c	0.39	0.11	0.1	0.09
18:1ω7c	0.53	0.24	0.08	0.08
18:1ω5c	0.41	0.12	0.01	0.01
18:00	0.37	0.09	0.07	0.06
10Me-18:1ω7c	0.51	0.23	0.01	0.01
10Me-18:0	0.29	0.04	0.11	0.07
cy19:0ω7c	0.32	0.06	0.05	0.03

Table S3. Characteristics of USCγ genomes from Chang Cave.

The information of USCγ MAGs retrieved from Chang Cave sediments.

Bin ID	Completeness (%)	Contamination (%)	Genome Size	Number of Contigs	Number of Coding Sequences	GC Content (%)	GTDB-Tk Classification	Accession Number
bin.9	90.38	5.29	2771219	317	317	60	p_Pseudomonadota; c_Gammaproteobacteria; o_JACCXJ01; f_JACCXJ01; g_USCg-Taylor	OEZ00020960
bin.10	92.61	0.85	3085996	315	315	58	p_Pseudomonadota; c_Gammaproteobacteria	OEZ00020962
bin.15	73.04	2.77	1968515	699	699	66	p_Pseudomonadota; c_Gammaproteobacteria; o_JACCXJ01; f_JACCXJ01; g_JACCXJ01	OEZ00020959
bin.24	90.55	0.71	2621274	321	321	58	p_Pseudomonadota; c_Gammaproteobacteria	OEZ00020961
bin.55	81.48	2.98	2680037	499	499	60	p_Pseudomonadota; c_Gammaproteobacteria	OEZ00020958

Completeness, contamination, genome size, number of contigs, number of coding sequences, and GC content were assessed with CheckM2 (v1.0.2). Phylogenetic assignments were generated using GTDB-Tk (v2.4.0) with default settings. All MAGs have been deposited in the NODE database (https://www.biosino.org/node/project/detail/OEP00005955) under the accession numbers listed.

Table S4. Amino acid identity comparisons among USCγ MAGs

Pairwise comparison of amino acid identity between MAGs from JACCXJ01 and USCγ MAGs from Chang Cave.

	**bin.15**	**bin.10**	**bin.9**	MSU_bin15	**bin.24**	MGR_bin175	MOJ_bin50	USCg_Taylor	**bin.55**	MOJ_bin5
**bin.15**	100	62.5	66.4	91.1	62.4	91.5	67.1	67.4	66.6	95.2
**bin.10**	61.18	100	62.9	62.6	99.2	62.4	63.8	63.3	63.3	62.9
**bin.9**	65.1	62.0	100	66.8	62.8	66.6	92.9	92.1	99.2	67.1
MSU_bin15	90.1	61.4	65.9	100	62.8	99.6	67.4	66.7	66.8	90.2
**bin.24**	60.8	99.2	61.6	61.9	100	62.6	63.2	62.9	62.9	62.9
MGR_bin175	90.9	61.2	65.8	99.6	61.3	100	67.2	66.8	66.6	90.5
MOJ_bin50	66.0	62.8	92.5	66.7	62.1	66.5	100	91.6	92.1	68.5
USCg_Taylor	65.9	62.6	91.8	65.8	62.2	66.0	91.2	100	91.2	68.0
**bin.55**	64.7	62.0	99.2	65.7	61.6	65.6	91.4	90.8	100	67.4
MOJ_bin5	94.8	61.5	66.3	90.3	61.5	90.3	68.0	66.9	66.4	100

The amino acid identity of MAGs from the order JACCXJ01 (GTDB) and USCγ MAGs (bold), recovered from the metagenome of Chang Cave sediment, was evaluated using two software tools: CompareM v0.1.2 (lower left) and EzAAI v1.2.3 (upper right).

Table S5. Differential expression of oxidative phosphorylation genes.

Differential expression of oxidative phosphorylation genes in MAGs (non-redundant gene set) under 500 ppm vs 2 ppm CH_4.

Gene identifier	baseMean	log_2FoldChange	lfcSE	stat	*P* value	adjusted *P* value	Gene annotation
k151_2020467_6	51.55	2.03	0.47	4.30	1.71E-05	0.001	*nuoF*
k151_2324101_4	37.41	2.08	0.53	3.90	9.82E-05	0.004	*sucA*
k151_4939623_2	90.95	1.23	0.32	3.79	1.49E-04	0.005	*atpA*
k127_1363749_2	61.81	1.59	0.42	3.77	1.62E-04	0.005	*atpG*
k151_4939623_3	37.30	1.66	0.51	3.24	1.18E-03	0.025	*atpG*
k127_1363749_1	30.32	2.11	0.65	3.22	1.28E-03	0.026	*atpA*
k127_4695773_1	62.13	1.22	0.39	3.10	1.91E-03	0.033	*coxB*

Data S1. (separate file)

Summary of Methane Oxidation Rates Across Habitat Types

REFERENCES

1. P. Forster, V. Ramaswamy, P. Artaxo, T. Berntsen, R. Betts, D. W. Fahey, J. Haywood, J. Lean, D. C. Lowe, G. Myhre, J. Nganga, R. Prinn, G. Raga, M. Schulz, V. R. Dorland, "Changes in atmospheric constituents and in radiative forcing" in *Climate Change 2007: The Physical Science Basis. Contribution of Working Group I to the Fourth Assessment Report of the Intergovernmental Panel on Climate Change*, S. Solomon, D. Qin, M. Manning, Z. Chen, M. Marquis, K. B. Averyt, M. Tignor, H. L. Miller, Eds. (Cambridge Univ. Press, 2007), pp. 129–234.

2. E. Nisbet, R. Fisher, D. Lowry, J. France, G. Allen, S. Bakkaloglu, T. Broderick, M. Cain, M. Coleman, J. Fernandez, Methane mitigation: Methods to reduce emissions, on the path to the Paris agreement. *Rev. Geophys.* **58**, e2019RG000675 (2020).

3. H. Tian, C. Lu, P. Ciais, A. M. Michalak, J. G. Canadell, E. Saikawa, D. N. Huntzinger, K. R. Gurney, S. Sitch, B. Zhang, J. Yang, P. Bousquet, L. Bruhwiler, G. Chen, E. Dlugokencky, P. Friedlingstein, J. Melillo, S. Pan, B. Poulter, R. Prinn, M. Saunois, C. R. Schwalm, S. C. Wofsy, The terrestrial biosphere as a net source of greenhouse gases to the atmosphere. *Nature* **531**, 225–228 (2016).

4. S. Kirschke, P. Bousquet, P. Ciais, M. Saunois, J. G. Canadell, E. J. Dlugokencky, P. Bergamaschi, D. Bergmann, D. R. Blake, L. Bruhwiler, Three decades of global methane sources and sinks. *Nat. Geosci.* **6**, 813–823 (2013).

5. X. Ni, P. M. Groffman, Declines in methane uptake in forest soils. *Proc. Natl. Acad. Sci. U.S.A.* **115**, 8587–8590 (2018).

6. D. P. Mattey, R. Fisher, T. C. Atkinson, J. P. Latin, R. Durrell, M. Ainsworth, D. Lowry, I. J. Fairchild, Methane in underground air in Gibraltar karst. *Earth Planet. Sci. Lett.* **374**, 71–80 (2013).

7. A. Fernandez-Cortes, S. Cuezva, M. Alvarez-Gallego, E. Garcia-Anton, C. Pla, D. Benavente, V. Jurado, C. Saiz-Jimenez, S. Sanchez-Moral, Subterranean atmospheres may act as daily methane sinks. *Nat. Commun.* **6**, 7003 (2015).

8. K. D. Webster, A. Drobniak, G. Etiope, M. Mastalerz, P. E. Sauer, A. Schimmelmann, Subterranean karst environments as a global sink for atmospheric methane. *Earth Planet. Sci. Lett.* **485**, 9–18 (2018).

9. L. K. McDonough, C. P. Iverach, S. Beckmann, M. Manefield, G. C. Rau, A. Baker, B. F. J. Kelly, Spatial variability of cave-air carbon dioxide and methane concentrations and isotopic compositions in a semi-arid karst environment. *Environ. Earth Sci.* **75**, 1–20 (2016).

10. K. D. Webster, A. Mirza, J. M. Deli, P. E. Sauer, A. Schimmelmann, Consumption of atmospheric methane in a limestone cave in Indiana, USA. *Chem. Geol.* **443**, 1–9 (2016).

11. C. L. Waring, S. I. Hankin, D. W. Griffith, M. A. Kertesz, V. Kobylski, N. L. Wilson, N. V. Coleman, G. Kettlewell, R. Zlot, M. Bosse, Seasonal total methane depletion in limestone caves. *Sci. Rep.* **7**, 8314 (2017).

12. J. T. Lennon, D. Nguyễn-Thùy, T. M. Phạm, A. Drobniak, P. Tạ, N. Phạm, T. Streil, K. Webster, A. Schimmelmann, Microbial contributions to subterranean methane sinks. *Geobiology* **15**, 254–258 (2017).

13. D. Nguyễn-Thuỳ, A. Schimmelmann, H. Nguyễn-Văn, A. Drobniak, J. T. Lennon, P. H. Tạ, N. T. Á. Nguyễn, Subterranean microbial oxidation of atmospheric methane in cavernous tropical karst. *Chem. Geol.* **466**, 229–238 (2017).

14. R. Zhao, H. Wang, X. Cheng, Y. Yun, X. Qiu, Upland soil cluster γ dominates the methanotroph communities in the karst Heshang Cave. *FEMS Microbiol. Ecol.* **94**, fiy192 (2018).

15. A. T. Tveit, A. G. Hestnes, S. L. Robinson, A. Schintlmeister, S. N. Dedysh, N. Jehmlich, M. von Bergen, C. Herbold, M. Wagner, A. Richter, M. M. Svenning, Widespread soil bacterium that oxidizes atmospheric methane. *Proc. Natl. Acad. Sci. U.S.A.* **116**, 8515–8524 (2019).

16. T. Schmider, A. G. Hestnes, J. Brzykcy, H. Schmidt, A. Schintlmeister, B. R. Roller, E. J. Teran, A. Söllinger, O. Schmidt, M. F. Polz, Physiological basis for atmospheric methane oxidation and methanotrophic growth on air. *Nat. Commun.* **15**, 4151 (2024).

17. C. Lüke, P. Frenzel, Potential of *pmoA* amplicon pyrosequencing for methanotroph diversity studies. *Appl. Environ. Microbiol.* **77**, 6305–6309 (2011).

18. C. Knief, Diversity and habitat preferences of cultivated and uncultivated aerobic methanotrophic bacteria evaluated based on *pmoA* as molecular marker. *Front. Microbiol.* **6**, 1346 (2015).

19. C. Knief, A. Lipski, P. F. Dunfield, Diversity and activity of methanotrophic bacteria in different upland soils. *Appl. Environ. Microbiol.* **69**, 6703–6714 (2003).

20. Y. Deng, R. Che, F. Wang, R. Conrad, M. Dumont, J. Yun, Y. Wu, A. Hu, J. Fang, Z. Xu, X. Cui, Y. Wang, Upland Soil Cluster Gamma dominates methanotrophic communities in upland grassland soils. *Sci. Total Environ.* **670**, 826–836 (2019).

21. J. Täumer, S. Kolb, R. S. Boeddinghaus, H. Wang, I. Schoning, M. Schrumpf, T. Urich, S. Marhan, Divergent drivers of the microbial methane sink in temperate forest and grassland soils. *Glob. Chang. Biol.* **27**, 929–940 (2021).

22. K. D. Webster, A. Schimmelmann, A. Drobniak, M. Mastalerz, L. Rosales Lagarde, P. J. Boston, J. T. Lennon, Diversity and composition of methanotroph communities in caves. *Microbiol. Spectrum* **10**, e0156621 (2022).

23. X. Cheng, X. Liu, H. Wang, C. Su, R. Zhao, P. L. E. Bodelier, W. Wang, L. Ma, X. Lu, J. A. Gralnick, USCγ dominated community composition and cooccurrence network of methanotrophs and bacteria in subterranean karst caves. *Microbiol. Spectrum* **9**, 10–1128 (2021).

24. C. R. Edwards, T. C. Onstott, J. M. Miller, J. B. Wiggins, W. Wang, C. K. Lee, S. C. Cary, S. B. Pointing, M. C. Y. Lau, Draft genome sequence of uncultured upland soil cluster Gammaproteobacteria gives molecular insights into high-affinity methanotrophy. *Genome Announc.* **5**, 10–1128 (2017).

25. M. Bender, R. Conrad, Kinetics of CH_4 oxidation in oxic soils exposed to ambient air or high CH_4 mixing ratios. *FEMS Microbiol. Ecol.* **101**, 261–270 (1992).

26. S. K. Bay, G. Ni, R. Lappan, P. M. Leung, W. W. Wong, S. I. Ry Holland, N. Athukorala, K. S. Knudsen, Z. Fan, M. Kerou, S. Jain, O. Schmidt, V. Eate, D. A. Clarke, T. Jirapanjawat, A. Tveit, T. Featonby, S. White, N. White, M. A. McGeoch, C. M. Singleton, P. L. M. Cook, S. L. Chown, C. Greening, Microbial aerotrophy enables continuous primary production in diverse cave ecosystems. *Nat. Commun.* **16**, 10295 (2025).

27. K. T. Konstantinidis, J. M. Tiedje, Towards a genome-based taxonomy for prokaryotes. *J. Bacteriol.* **187**, 6258–6264 (2005).

28. C. Luo, L. M. Rodriguez-r, K. Konstantinidis, MyTaxa: An advanced taxonomic classifier for genomic and metagenomic sequences. *Nucleic Acids Res.* **42**, e73 (2014).

29. S. Yoon, J. Im, N. Bandow, A. A. DiSpirito, J. D. Semrau, Constitutive expression of pMMO by *Methylocystis* strain SB2 when grown on multi-carbon substrates: Implications for biodegradation of chlorinated ethenes. *Environ. Microbiol. Rep.* **3**, 182–188 (2011).

30. S. E. Belova, M. Baani, N. E. Suzina, P. L. Bodelier, W. Liesack, S. N. Dedysh, Acetate utilization as a survival strategy of peat-inhabiting *Methylocystis* spp. *Environ. Microbiol. Rep.* **3**, 36–46 (2011).

31. J. Pratscher, J. Vollmers, S. Wiegand, M. G. Dumont, A. K. Kaster, Unravelling the identity, metabolic potential and global biogeography of the atmospheric methane-oxidizing upland soil cluster α. *Environ. Microbiol.* **20**, 1016–1029 (2018).

32. T. Ferenci, Trade-off mechanisms shaping the diversity of bacteria. *Trends Microbiol.* **24**, 209–223 (2016).

33. M. Baani, W. Liesack, Two isozymes of particulate methane monooxygenase with different methane oxidation kinetics are found in *Methylocystis* sp. strain SC2. *Proc. Natl. Acad. Sci. U.S.A.* **105**, 10203–10208 (2008).

34. D. S. Reay, D. B. Nedwell, Methane oxidation in temperate soils: Effects of inorganic N. *Soil Biol. Biochem.* **36**, 2059–2065 (2004).

35. R. Conrad, Soil microorganisms as controllers of atmospheric trace gases (H_2, CO, CH_4, OCS, N_2O, and NO). *Microbiol. Rev.* **60**, 609–640 (1996).

36. G. M. King, “Ecophysiological characteristics of obligate methanotrophic bacteria and methane oxidation in situ” in *Microbial Growth on C1 Compounds*, J. C. Murrell, D. P. Kelly, Eds. (Intercept, 1993), pp. 303–313.

37. P. F. Dunfield, “The soil methane sink” in *Greenhouse Gas Sinks*, D. S. Reay, C. N. Hewitt, K. A. Smith, J. Grace, Eds. (CAB International, 2007), pp. 152–170.

38. R. Henneberger, E. Chiri, P. E. Bodelier, P. Frenzel, C. Luke, M. H. Schroth, Field-scale tracking of active methane-oxidizing communities in a landfill cover soil reveals spatial and seasonal variability. *Environ. Microbiol.* **17**, 1721–1737 (2015).

39. T. Stötter, D. Bastviken, P. L. Bodelier, M. van Hardenbroek, P. Rinta, J. Schilder, C. J. Schubert, O. Heiri, Abundance and $\delta^{13}C$ values of fatty acids in lacustrine surface sediments: Relationships with in-lake methane concentrations. *Quat. Sci. Rev.* **191**, 337–347 (2018).

40. N. Sultana, J. Zhao, Y. Zheng, Y. Cai, M. Faheem, X. Peng, W. Wang, Z. Jia, Stable isotope probing of active methane oxidizers in rice field soils from cold regions. *Biol. Fertil. Soils* **55**, 243–250 (2019).

41. N. Sultana, Z. Jun, C. Yuanfeng, G. M. Rahman, M. S. Alam, M. Faheem, H. Adrian, J. Zhongjun, Methanotrophy-driven accumulation of organic carbon in four paddy soils of Bangladesh. *Pedosphere* **32**, 348–358 (2022).

42. G. Börjesson, I. Sundh, A. Tunlid, Å. Frostegård, B. H. Svensson, Microbial oxidation of CH_4 at high partial pressures in an organic landfill cover soil under different moisture regimes. *FEMS Microbiol. Ecol.* **26**, 207–217 (1998).

43. P. Roslev, N. Iversen, K. Henriksen, Oxidation and assimilation of atmospheric methane by soil methane oxidizers. *Appl. Environ. Microbiol.* **63**, 874–880 (1997).

44. S. Whalen, W. Reeburgh, Consumption of atmospheric methane by tundra soils. *Nature* **346**, 160–162 (1990).

45. R. Conrad, "Capacity of aerobic microorganisms to utilize and grow on atmospheric trace gases (H_2, CO, CH_4)" in *Current Perspectives in Microbial Ecology*, M. J. Klug, C. A. Reddy, Eds. (American Society for Microbiology, 1984), pp. 461–467.

46. J. B. Russell, G. M. Cook, Energetics of bacterial growth: Balance of anabolic and catabolic reactions. *Microbiol. Rev.* **59**, 48–62 (1995).

47. A. Lawrence, J. Quayle, Alternative carbon assimilation pathways in methane-utilizing bacteria. *Microbiology* **63**, 371–374 (1970).

48. X. Yang, Q. Yuan, H. Luo, F. Li, Y. Mao, X. Zhao, J. Du, P. Li, X. Ju, Y. Zheng, Systematic design and in vitro validation of novel one-carbon assimilation pathways. *Metab. Eng.* **56**, 142–153 (2019).

49. W.-C. Kao, Y.-R. Chen, C. Y. Eugene, H. Lee, Q. Tian, K.-M. Wu, S.-F. Tsai, S. S.-F. Yu, Y.-J. Chen, R. Aebersold, Quantitative proteomic analysis of metabolic regulation by copper ions in *Methylococcus capsulatus* (Bath). *J. Biol. Chem.* **279**, 51554–51560 (2004).

50. M. Kalyuzhnaya, S. Yang, O. Rozova, N. Smalley, J. Clubb, A. Lamb, G. N. Gowda, D. Raftery, Y. Fu, F. Bringel, Highly efficient methane biocatalysis revealed in a methanotrophic bacterium. *Nat. Commun.* **4**, 2785 (2013).

51. H. Yu, J. C. Liao, A modified serine cycle in *Escherichia coli* coverts methanol and CO_2 to two-carbon compounds. *Nat. Commun.* **9**, 3992 (2018).

52. H. Šmejkalová, T. J. Erb, G. Fuchs, Methanol assimilation in *Methylobacterium extorquens* AM1: Demonstration of all enzymes and their regulation. *PLOS ONE* **5**, e13001 (2010).

53. G. J. Crowther, G. Kosály, M. E. Lidstrom, Formate as the main branch point for methylotrophic metabolism in *Methylobacterium extorquens* AM1. *J. Bacteriol.* **190**, 5057–5062 (2008).

54. S. C. Taylor, H. Dalton, C. S. Dow, Ribulose-1, 5-bisphosphate carboxylase/oxygenase and carbon assimilation in *Methylococcus capsulatus* (Bath). *Microbiology* **122**, 89–94 (1981).

55. O. Rasigraf, D. M. Kool, M. S. Jetten, J. S. Sinninghe Damsté, K. F. Ettwig, Autotrophic carbon dioxide fixation via the Calvin-Benson-Bassham cycle by the denitrifying methanotroph "*Candidatus* Methylomirabilis oxyfera". *Appl. Environ. Microbiol.* **80**, 2451–2460 (2014).

56. A. F. Khadem, A. Pol, A. Wieczorek, S. S. Mohammadi, K.-J. Francoijs, H. G. Stunnenberg, M. S. Jetten, H. J. Op den Camp, Autotrophic methanotrophy in Verrucomicrobia: *Methylacidiphilum fumariolicum* SolV uses the Calvin-Benson-Bassham cycle for carbon dioxide fixation. *J. Bacteriol.* **193**, 4438–4446 (2011).

57. A. T. Tveit, T. Schmider, A. G. Hestnes, M. Lindgren, A. Didriksen, M. M. Svenning, Simultaneous oxidation of atmospheric methane, carbon monoxide and hydrogen for bacterial growth. *Microorganisms* **9**, 153 (2021).

58. M. Aragno, "Thermophilic, aerobic, hydrogen-oxidizing (Knallgas) bacteria" in *The Prokaryotes*, A. Balows, H. G. Trüper, M. Dworkin, W. Harder, K.-H. Schleifer, Eds. (Springer, 1992), pp. 3917–3933.

59. S. Mohammadi, A. Pol, T. A. van Alen, M. S. Jetten, H. J. Op den Camp, *Methylacidiphilum fumariolicum* SolV, a thermoacidophilic 'Knallgas' methanotroph with both an oxygen-sensitive and -insensitive hydrogenase. *ISME J.* **11**, 945–958 (2017).

60. C. R. Carere, K. Hards, K. M. Houghton, J. F. Power, B. McDonald, C. Collet, D. J. Gapes, R. Sparling, E. S. Boyd, G. M. Cook, C. Greening, M. B. Stott, Mixotrophy drives niche expansion of verrucomicrobial methanotrophs. *ISME J.* **11**, 2599–2610 (2017).

61. S. Mammola, E. Piano, P. Cardoso, P. Vernon, D. Domínguez-Villar, D. C. Culver, T. Pipan, M. Isaia, Climate change going deep: The effects of global climatic alterations on cave ecosystems. *Anthropocene Rev.* **6**, 98–116 (2019).

62. M. Barthel, M. Bauters, S. Baumgartner, T. W. Drake, N. M. Bey, G. Bush, P. Boeckx, C. I. Botefa, N. Dériaz, G. L. Ekamba, Low N_2O and variable CH_4 fluxes from tropical forest soils of the Congo Basin. *Nat. Commun.* **13**, 330 (2022).

63. H. Song, C. Peng, Q. Zhu, Z. Chen, J.-P. Blanchet, Q. Liu, T. Li, P. Li, Z. Liu, Quantification and uncertainty of global upland soil methane sinks: Processes, controls, model limitations, and improvements. *Earth Sci. Rev.* **252**, 104758 (2024).

64. FAO, *Global Forest Resources Assessment 2020: Main Report* (Food and Agricultural Organization of the United Nations, 2020); www.scirp.org/reference/referencespapers?referenceid=3337671.

65. R. P. White, S. Murray, M. Rohweder, S. Prince, K. Thompson, *Pilot Analysis of Global Ecosystems: Grassland Ecosystems* (World Resources Institute, 2000).

66. N. Goldscheider, Z. Chen, A. S. Auler, M. Bakalowicz, S. Broda, D. Drew, J. Hartmann, G. Jiang, N. Moosdorf, Z. Stevanovic, G. Veni, Global distribution of carbonate rocks and karst water resources. *Hydrgeol. J.* **28**, 1661–1677 (2020).

67. Y. Wang, H. Chen, Q. Zhu, C. Peng, N. Wu, G. Yang, D. Zhu, J. Tian, L. Tian, X. Kang, Soil methane uptake by grasslands and forests in China. *Soil Biol. Biochem.* **74**, 70–81 (2014).

68. D. Yang, Y. Gao, J. Zhao, Y. Xue, W. Zhang, W. Wu, H. Jiang, D. Cao, Agricultural methane emissions in China: Inventories, driving forces and mitigation strategies. *Environ. Sci. Technol.* **57**, 13292–13303 (2023).

69. L. Michaelis, M. L. Menten, The kinetics of invertase action. *Biochem. Z.* **49**, 352 (1913).

70. D. A. Wiesenburg, N. L. Guinasso Jr., Equilibrium solubilities of methane, carbon monoxide, and hydrogen in water and sea water. *J. Chem. Eng. Data* **24**, 356–360 (1979).

71. X. Wu, M. Pan, J. Yin, J. Cao, Hydrogen and oxygen isotope signal transmission in rainfall, soil water, and cave drip water in Liangfeng Cave, Southwest China. *Appl. Geochem.* **158**, 105798 (2023).

72. B. Chang, J. Huang, T. J. Algeo, R. D. Pancost, X. Wan, Y. Xue, J. Jia, Z. Wang, J. Hu, J. Wang, Episodic massive release of methane during the mid-Cretaceous greenhouse. *Geol. Soc. Am. Bull.* **134**, 2958–2970 (2022).

73. J. Liu, J. Li, L. Feng, H. Cao, Z. Cui, An improved method for extracting bacteria from soil for high molecular weight DNA recovery and BAC library construction. *J. Microbiol.* **48**, 728–733 (2011).

74. D. Liu, H. Dong, L. Zhao, H. Wang, Smectite reduction by *Shewanella* species as facilitated by cystine and cysteine. *Geomicrobiol. J.* **31**, 53–63 (2013).

75. E. G. Bligh, W. J. Dyer, A rapid method of total lipid extraction and purification. *Can. J. Biochem. Physiol.* **37**, 911–917 (1959).

76. D. White, W. Davis, J. Nickels, J. King, R. Bobbie, Determination of the sedimentary microbial biomass by extractible lipid phosphate. *Oecologia* **40**, 51–62 (1979).

77. Y. Zhang, B. D. A. Naafs, X. Huang, Q. Song, J. Xue, R. Wang, M. Zhao, R. P. Evershed, R. D. Pancost, S. Xie, Variations in wetland hydrology drive rapid changes in the microbial community, carbon metabolic activity, and greenhouse gas fluxes. *Geochim. Cosmochim. Acta* **317**, 269–285 (2022).

78. N. J. Jensen, M. L. Gross, Mass spectrometry methods for structural determination and analysis of fatty acids. *Mass Spectrom. Rev.* **6**, 497–536 (1987).

79. P. D. Nichols, J. B. Guckert, D. C. White, Determination of monosaturated fatty acid double-bond position and geometry for microbial monocultures and complex consortia by capillary GC-MS of their dimethyl disulphide adducts. *J. Microbiol. Methods* **5**, 49–55 (1986).

80. D. G. Bourne, I. R. McDonald, J. C. Murrell, Comparison of *pmoA* PCR primer sets as tools for investigating methanotroph diversity in three Danish soils. *Appl. Environ. Microbiol.* **67**, 3802–3809 (2001).

81. P. D. Schloss, S. L. Westcott, T. Ryabin, J. R. Hall, M. Hartmann, E. B. Hollister, R. A. Lesniewski, B. B. Oakley, D. H. Parks, C. J. Robinson, J. W. Sahl, B. Stres, G. G. Thallinger, D. van Horn, C. F. Weber, Introducing mothur: Open-source, platform-independent, community-supported software for describing and comparing microbial communities. *Appl. Environ. Microbiol.* **75**, 7537–7541 (2009).

82. T. Rognes, T. Flouri, B. Nichols, C. Quince, F. Mahé, VSEARCH: A versatile open source tool for metagenomics. *PeerJ Life Environ.* **4**, e2584 (2016).

83. Q. Wang, J. F. Quensen III, J. A. Fish, T. Kwon Lee, Y. Sun, J. M. Tiedje, J. R. Cole, Ecological patterns of *nifH* genes in four terrestrial climatic zones explored with targeted metagenomics using FrameBot, a new informatics tool. *MBio* **4**, 10–1128 (2013).

84. D. V. Prasad, S. Madhusudanan, S. Jaganathan, uCLUST-a new algorithm for clustering unstructured data. *ARPN J. Eng. Appl. Sci.* **10**, 2108–2117 (2015).

85. R. C. Edgar, Search and clustering orders of magnitude faster than BLAST. *Bioinformatics* **26**, 2460–2461 (2010).

86. Z. Zhang, W. Miller, D. J. Lipman, Gapped BLAST and PSI-BLAST: A new generation of protein database search programs. *Nucleic Acids Res.* **25**, 3389–3402 (1997).

87. E. Chiri, P. A. Nauer, E.-M. Rainer, J. Zeyer, M. H. Schroth, High temporal and spatial variability of atmospheric-methane oxidation in alpine glacier forefield soils. *Appl. Environ. Microbiol.* **83**, e01139–e01117 (2017).

88. C. Martineau, Y. Pan, L. Bodrossy, E. Yergeau, L. G. Whyte, C. W. Greer, Atmospheric methane oxidizers are present and active in Canadian high Arctic soils. *FEMS Microbiol. Ecol.* **89**, 257–269 (2014).

89. S. Kolb, C. Knief, P. F. Dunfield, R. Conrad, Abundance and activity of uncultured methanotrophic bacteria involved in the consumption of atmospheric methane in two forest soils. *Environ. Microbiol.* **7**, 1150–1161 (2005).

90. S. Kolb, C. Knief, S. Stubner, R. Conrad, Quantitative detection of methanotrophs in soil by novel *pmoA*-targeted real-time PCR assays. *Appl. Environ. Microbiol.* **69**, 2423–2429 (2003).

91. M. Martin, Cutadapt removes adapter sequences from high-throughput sequencing reads. *EMBnet J.* **17**, 10–12 (2011).

92. D. Li, C.-M. Liu, R. Luo, K. Sadakane, T.-W. Lam, MEGAHIT: An ultra-fast single-node solution for large and complex metagenomics assembly via succinct de Bruijn graph. *Bioinformatics* **31**, 1674–1676 (2015).

93. G. V. Uritskiy, J. DiRuggiero, J. Taylor, MetaWRAP—A flexible pipeline for genome-resolved metagenomic data analysis. *Microbiome* **6**, 158 (2018).

94. D. H. Parks, M. Imelfort, C. T. Skennerton, P. Hugenholtz, G. W. Tyson, CheckM: Assessing the quality of microbial genomes recovered from isolates, single cells, and metagenomes. *Genome Res.* **25**, 1043–1055 (2015).

95. P.-A. Chaumeil, A. J. Mussig, P. Hugenholtz, D. H. Parks, GTDB-Tk: A toolkit to classify genomes with the Genome Taxonomy Database. *Bioinformatics* **36**, 1925–1927 (2019).

96. L.-T. Nguyen, H. A. Schmidt, A. Von Haeseler, B. Q. Minh, IQ-TREE: A fast and effective stochastic algorithm for estimating maximum-likelihood phylogenies. *Mol. Biol. Evol.* **32**, 268–274 (2015).

97. A. Chklovski, D. H. Parks, B. J. Woodcroft, G. W. Tyson, CheckM2: A rapid, scalable and accurate tool for assessing microbial genome quality using machine learning. *Nat. Methods* **20**, 1203–1212 (2023).

98. K. Katoh, K. Misawa, K. I. Kuma, T. Miyata, MAFFT: A novel method for rapid multiple sequence alignment based on fast Fourier transform. *Nucleic Acids Res.* **30**, 3059–3066 (2002).

99. S. Capella-Gutiérrez, J. M. Silla-Martínez, T. Gabaldón, trimAl: A tool for automated alignment trimming in large-scale phylogenetic analyses. *Bioinformatics* **25**, 1972–1973 (2009).

100. M. Shaffer, M. A. Borton, B. B. McGivern, A. A. Zayed, S. L. La Rosa, L. M. Solden, P. Liu, A. B. Narrowe, J. Rodríguez-Ramos, B. Bolduc, DRAM for distilling microbial metabolism to automate the curation of microbiome function. *Nucleic Acids Res.* **48**, 8883–8900 (2020).

101. Z. Zhou, P. Q. Tran, A. M. Breister, Y. Liu, K. Kieft, E. S. Cowley, U. Karaoz, K. Anantharaman, METABOLIC: High-throughput profiling of microbial genomes for functional traits, metabolism, biogeochemistry, and community-scale functional networks. *Microbiome* **10**, 33 (2022).

102. C. P. Cantalapiedra, A. Hernández-Plaza, I. Letunic, P. Bork, J. Huerta-Cepas, eggNOG-mapper v2: Functional annotation, orthology assignments, and domain prediction at the metagenomic scale. *Mol. Biol. Evol.* **38**, 5825–5829 (2021).

103. M. Kanehisa, "The KEGG database" in *In silico simulation of biological processes: Novartis Foundation Symposium*, vol. 247 (John Wiley & Sons, 2002), pp. 91–103.

104. D. Kim, S. Park, J. Chun, Introducing EzAAI: A pipeline for high throughput calculations of prokaryotic average amino acid identity. *J. Microbiol.* **59**, 476–480 (2021).

105. S. Chen, Y. Zhou, Y. Chen, J. Gu, fastp: An ultra-fast all-in-one FASTQ preprocessor. *Bioinformatics* **34**, i884–i890 (2018).

106. E. Kopylova, L. Noé, H. Touzet, SortMeRNA: Fast and accurate filtering of ribosomal RNAs in metatranscriptomic data. *Bioinformatics* **28**, 3211–3217 (2012).

107. M. Szymanski, A. Zielezinski, J. Barciszewski, V. A. Erdmann, W. M. Karlowski, 5SRNAdb: An information resource for 5S ribosomal RNAs. *Nucleic Acids Res.* **44**, D180–D183 (2016).

108. C. Quast, E. Pruesse, P. Yilmaz, J. Gerken, T. Schweer, P. Yarza, J. Peplies, F. O. Glöckner, The SILVA ribosomal RNA gene database project: Improved data processing and web-based tools. *Nucleic Acids Res.* **41**, D590–D596 (2012).

109. B. J. Haas, A. Papanicolaou, M. Yassour, M. Grabherr, P. D. Blood, J. Bowden, M. B. Couger, D. Eccles, B. Li, M. Lieber, M. MacManes, M. Ott, J. Orvis, N. Pochet, F. Strozzi, N. Weeks, R. Westerman, T. William, C. N. Dewey, R. Henschel, R. LeDuc, N. Friedman, A. Regev, De novo transcript sequence reconstruction from RNA-seq using the Trinity platform for reference generation and analysis. *Nat. Protoc.* **8**, 1494–1512 (2013).

110. B. Li, C. N. Dewey, RSEM: Accurate transcript quantification from RNA-seq data with or without a reference genome. *BMC Bioinf.* **12**, 1–16 (2011).

111. S. T. Aroney, R. J. Newell, J. N. Nissen, A. P. Camargo, G. W. Tyson, B. J. Woodcroft, CoverM: Read alignment statistics for metagenomics. *Bioinformatics* **41**, btaf147 (2025).

112. B. Li, V. Ruotti, R. M. Stewart, J. A. Thomson, C. N. Dewey, RNA-seq gene expression estimation with read mapping uncertainty. *Bioinformatics* **26**, 493–500 (2010).

113. G. Zeng, W. Lu, Y. Wang, H. Peng, P. Chen, X. Weng, J. Chen, L. Zhang, H. Du, W. Luo, S. Wang, Methane sink of subterranean space in an integrated atmosphere-soil-cave system. *Environ. Res.* **252**, 118904 (2024).

114. I. Lagkouvardos, D. Joseph, M. Kapfhammer, S. Giritli, M. Horn, D. Haller, T. Clavel, IMNGS: A comprehensive open resource of processed 16*S* rRNA microbial profiles for ecology and diversity studies. *Sci. Rep.* **6**, 33721 (2016).

115. E. Pruesse, J. Peplies, F. O. Glöckner, SINA: Accurate high-throughput multiple sequence alignment of ribosomal RNA genes. *Bioinformatics* **28**, 1823–1829 (2012).

116. S. A. Berger, D. Krompass, A. Stamatakis, Performance, accuracy, and web server for evolutionary placement of short sequence reads under maximum likelihood. *Syst. Biol.* **60**, 291–302 (2011).

117. D. Ford, P. D. Williams, *Karst Hydrogeology and Geomorphology* (John Wiley & Sons, 2007); 10.1002/9781118684986.
